# Inhibition of Mitochondrial Fission Reverses Simulated Microgravity-Induced Osteoblast Dysfunction by Enhancing Mechanotransduction and Epigenetic Modification

**DOI:** 10.34133/research.0602

**Published:** 2025-02-04

**Authors:** Qiusheng Shi, Yaxin Song, Jingqi Cao, Jing Na, Zhijie Yang, Xinyuan Chen, Ziyi Wang, Yubo Fan, Lisha Zheng

**Affiliations:** Key Laboratory of Biomechanics and Mechanobiology (Beihang University), Ministry of Education, Beijing Advanced Innovation Center for Biomedical Engineering, School of Biological Science and Medical Engineering, Beihang University, Beijing 100083, China.

## Abstract

Simulated microgravity (SMG) poses substantial challenges to astronaut health, particularly impacting osteoblast function and leading to disuse osteoporosis. This study investigates the adverse effects of SMG on osteoblasts, focusing on changes in mitochondrial dynamics and their consequent effects on cellular energy metabolism and mechanotransduction pathways. We discovered that SMG markedly reduced the expression of osteoblast differentiation markers and promoted mitochondrial fission, as indicated by an increase in punctate mitochondria, a decrease in mitochondrial length, and a reduction in cristae density. These mitochondrial alterations are linked to elevated reactive oxygen species levels, a decrease in ΔΨm, and a metabolic shift from oxidative phosphorylation to glycolysis, resulting in decreased adenosine triphosphate production, which are all indicative of mitochondrial dysfunction. Our results showed that treatment with mitochondrial division inhibitor-1 (mdivi-1), a mitochondrial fission inhibitor, effectively inhibited these SMG-induced effects, thereby maintaining mitochondrial structure and function and promoting osteoblast differentiation. Furthermore, SMG disrupted critical mechanotransduction processes, by affecting paxillin expression, the RhoA–ROCK–Myosin II pathway, and actin dynamics, which subsequently altered nuclear morphology and disrupted Yes-associated protein signaling. Notably, treatment with mdivi-1 prevented these disruptions in mechanotransduction pathways. Moreover, our study showed that SMG-induced chromatin remodeling and histone methylation, which are epigenetic barriers to osteogenic differentiation, can be reversed by targeting mitochondrial fission, further highlighting the significance of mitochondrial dynamics in osteoblast function in an SMG environment. Therefore, targeting mitochondrial fission emerges as a promising therapeutic strategy to alleviate osteoblast dysfunction under SMG conditions, providing novel approaches to maintain bone health during prolonged space missions and safeguard the astronaut well-being.

## Introduction

Microgravity substantially impacts bone health by decreasing bone mineral density (BMD), leading to bone loss during prolonged spaceflights [1]. Documented as early as the mid-1970s with the Skylab missions, the decrease in BMD at a rate of 1% to 2% per month highlights a persistent and unresolved challenge in human space exploration [2,3]. Despite advancements in countermeasures, including rigorous exercise routines and dietary enhancements with calcium and vitamin D [4,5], bone loss in space remains a formidable obstacle, necessitating a deeper understanding of its underlying mechanisms to develop more effective interventions. Bone homeostasis, the balance between bone formation and bone resorption, is crucial for bone strength and integrity [6,7]. Previous studies have elucidated the accelerated bone loss experienced in microgravity environments, pinpointing osteoblast dysfunction as a primary contributor [8,9]. However, the cellular and molecular mechanisms mediating osteoblast dysfunction in microgravity are still not fully understood. Uncovering these mechanisms is key to developing better strategies against bone loss, benefiting astronauts and advancing osteoporosis treatments.

Osteoblastic bone formation is an energy-demanding process requiring substantial metabolic activity [10]. Mitochondria are central to cellular energy production and regulate various cellular functions, including calcium homeostasis, redox signaling, and autophagy [11,12]. Mitochondrial dynamics, characterized by a constant balance between fission and fusion processes, adapt to meet cellular energy requirements and are integral to the differentiation and matrix mineralization activities of osteoblasts [13,14]. Notably, mature osteoblasts have a predominantly fused mitochondrial morphology, engaging in oxidative phosphorylation (OXPHOS), which is essential for the deposition of collagen and the mineralization phase of bone formation [10,15]. Recent studies have shown that during prolonged spaceflights, astronauts experience marked disruptions in mitochondrial function due to microgravity conditions [16,17]. Specifically, microgravity leads to reduced mitochondrial energy production efficiency, resulting in lower adenosine triphosphate (ATP) synthesis. This decrease in energy supply adversely impacts osteoblast activity and their ability to mineralize bone matrix [18]. Consequently, this diminished metabolic function not only slows down bone formation but also potentially increases bone resorption rates, thereby contributing to the bone loss observed in astronauts during space missions. Therefore, exploring the effects of microgravity on the dynamics and function of mitochondria in osteoblasts, as well as its extensive impact on cellular functions, is crucial for elucidating the mechanisms of bone formation in microgravity.

Additionally, osteoblasts are sensitive to mechanical signals, which they translate into cellular responses through mechanotransduction pathways [19]. Central to this process are focal adhesions (FAs) on the cell membrane, which bind to extracellular matrix (ECM) proteins and facilitate cell–ECM interactions [20]. These FAs are critical for sensing mechanical stimuli from the ECM and triggering the production and relay of intracellular biochemical signals [21]. The activation of FAs induces the formation and stabilization of stress fibers within the cytoplasm, regulated by Ras homologue family member A (RhoA) GTPase, Rho-associated kinase (ROCK), and Myosin II [22,23]. Subsequently, ROCK and Myosin II activate the transcriptional activator Yes-associated protein (YAP), leading to its translocation to the nucleus [24]. YAP, highly sensitive to mechanical stimuli, plays a pivotal role in osteogenic differentiation, essential for skeletal development and bone repair [25]. Recent studies indicate that alterations in the physical and mechanical characteristics of the ECM can lead to metabolic changes through FA-mediated mechanosignaling [26,27]. However, the role of mitochondrial dynamics and metabolic homeostasis in the regulation of mechanotransduction in osteoblasts under simulated microgravity (SMG) conditions is still largely unexplored.

This study aims to bridge these gaps by examining the influence of mitochondrial dynamics on osteoblast function under SMG conditions. Focusing on mitochondrial fission and its modulation through the use of the mitochondrial division inhibitor-1 (mdivi-1), we investigate the role of mitochondrial dynamics in mediating the adverse effects of SMG on osteoblasts. By elucidating the connections between mitochondrial dysfunction, shifts in cellular metabolism, disruption of mechanotransduction pathways, and epigenetic modifications, this study aims to uncover novel therapeutic strategies to mitigate bone loss in microgravity, thereby contributing to the enhancement of astronaut health and the advancement of long-term space exploration.

## Results

### SMG-induced osteoblast dysfunction and mitochondrial fission

**Fig. 1.**
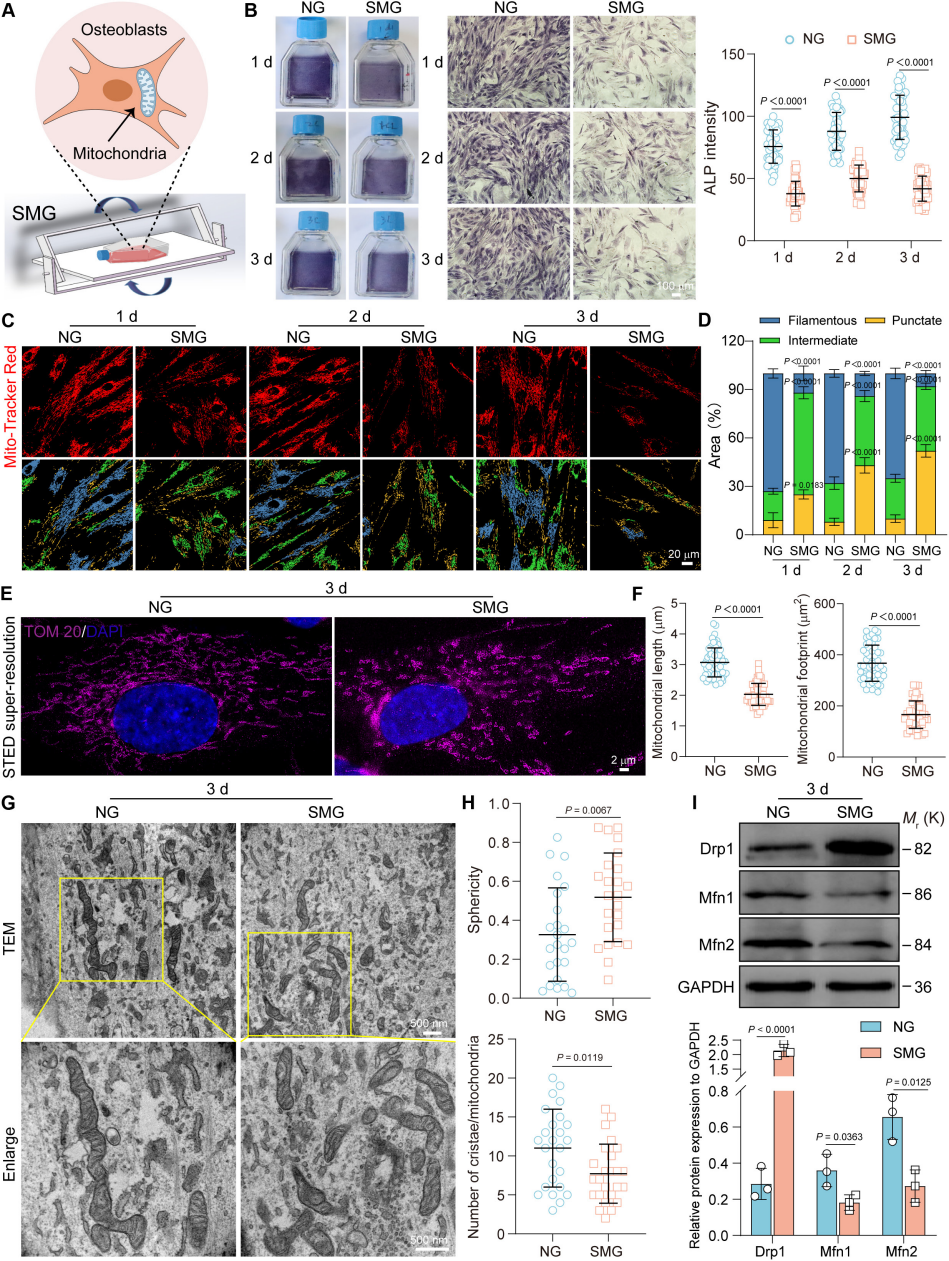
SMG-induced osteoblast dysfunction and mitochondrial fission. (A) Diagram of the SMG setup. (B) ALP staining and intensity altered after 1 to 3 days under NG or SMG conditions (*n* = 44 for each group); scale bar: 100 μm. (C) Representative immunofluorescent images show osteoblast mitochondria labeled with MitoTracker Red, classified by mitochondrial morphology, and displayed in different colors after 1 to 3 days under NG or SMG conditions; scale bar: 20 μm. (D) The classification was based on the area percentages of punctate, intermediate, and filamentous mitochondria (*n* = 15 for each group). (E) Super-resolution STED imaging reveals mitochondria labeled with TOM20 after 3 days of NG or SMG exposure; scale bars: 2 μm. (F) Metrics of mitochondrial length (*n* = 60 for each group) and footprint (*n* = 50 for each group). (G) TEM analysis of mitochondrial ultrastructure after 3 days of NG or SMG exposure. Yellow boxes indicate the regions that were shown as a magnified view; scale bars: 500 nm. (H) Quantitative assessment of mitochondrial sphericity and cristae number (*n* = 25 for each group). (I) Protein expression levels of Drp1, Mfn1, and Mfn2 after 3 days of NG or SMG exposure (*n* = 3). Results reflect ≥3 biological repeats, mean ± SD, *P* values via ANOVA or *t* test.

We investigated the effects of SMG on osteoblast differentiation using a 2-dimensional rotating system (Fig. 1A). Observations from 1 to 3 days of SMG exposure demonstrated significant reductions in the mRNA expression levels of *ALP*, *OPN*, and *RUNX2* (Fig. S1A), as well as marked decreases in the enzymatic activity and staining intensity of cellular ALP (Fig. 1B and Fig. S1B). These findings align with our previous observations of SMG-induced osteoblast dysfunction [9]. Given the critical role of mitochondrial dynamics in osteoblast function, we also examined the effects of SMG on these processes. Mitochondrial morphology was visualized using MitoTracker Red staining and classified as punctate, intermediate, or filamentous using the R Caret package, an advanced machine learning classification tool [28,29]. Exposure to SMG led to an increase in the area proportion of punctate and intermediate mitochondria, while decreasing the area proportion of filamentous forms (Fig. 1C and D). Further examination with stimulated emission depletion (STED) super-resolution microscopy, using TOM20 immunolabeling, highlighted significant reductions in mitochondrial length and footprint following exposure to SMG (Fig. 1E and F). Transmission electron microscopy (TEM) analysis also showed substantial changes in mitochondrial ultrastructure after 3 days of exposure to SMG, including transitions from elongated to more rounded morphology, increased sphericity, and a decrease in the number of cristae (Fig. 1G and H). Analysis of mitochondrial dynamics regulators revealed decreased levels of fusion proteins mitofusin 1 (Mfn1) and Mfn2 and an increase in the fission protein dynamin-related protein 1 (Drp1) (Fig. 1I), suggesting that exposure to SMG promotes mitochondrial fission. Overall, these results indicate that SMG disrupts osteoblast function while concurrently inducing mitochondrial fission.

### SMG increased oxidative stress and mitochondrial dysfunction in osteoblasts

**Fig. 2.**
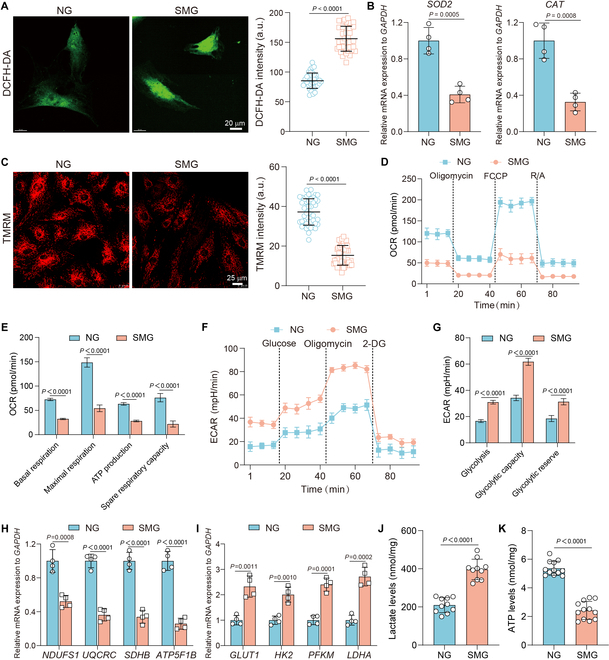
SMG increased oxidative stress and mitochondrial dysfunction in osteoblasts. (A) Visualization of intracellular ROS with DCFH-DA after 3 days of NG or SMG exposure and fluorescent intensity analysis of ROS (*n* = 37 for each group); scale bars: 20 μm. (B) Expression levels of antioxidant genes *SOD2* and *CAT* after 3 days of NG or SMG exposure (*n* ≥ 3). (C) ΔΨm assessed by TMRM staining after 3 days of NG or SMG exposure and quantification of ΔΨm fluorescent intensity (*n* = 45 for each group); scale bars: 25 μm. (D) Real-time measurements and quantifications of mitochondrial OCR in osteoblasts after 3 days of NG or SMG exposure; R/A: rotenone/antimycin A. (E) Basal respiration, maximal respiration, ATP production, and spare respiratory capacity were measured (*n* = 6 for each group). (F) Real-time measurements and quantifications of mitochondrial ECAR in osteoblasts after 3 days of NG or SMG exposure. (G) Glycolysis, glycolytic capacity, and glycolytic reserve were measured (*n* = 6 for each group). (H and I) RT-PCR analysis of oxidative phosphorylation family genes (*NDUFB8*, *UQCRC2*, *SDHB*, and *ATP5F1B*) and glycolysis family genes (*GLUT1*, *HK2*, *PFKM*, and *LDHA*) in osteoblasts after 3 days of NG or SMG exposure (*n* ≥ 3). (J and K) Lactate production (*n* = 10 for each group) and ATP levels (*n* = 12 for each group) after 3 days of NG or SMG exposure, normalized to total protein level. Results reflect ≥3 biological repeats, mean ± SD, *P* values via ANOVA or *t* test.

Disruption of mitochondrial dynamics can affect the activity of reactive oxygen species (ROS)-scavenging enzymatic activity and lead to increased ROS levels and mitochondrial respiratory chain complex dysfunction [30]. We measured intracellular ROS levels using the 2′,7′-dichlorodihydrofluorescein diacetate (DCFH-DA) assay and found that exposure of osteoblasts to SMG for 3 days markedly increased ROS production, as indicated by the significantly elevated DCFH-DA fluorescence intensity (Fig. 2A). Additionally, SMG-exposed osteoblasts showed considerable decreased mRNA expression levels of antioxidant enzymes, such as superoxide dismutase 2 (*SOD2*) and catalase (*CAT*) (Fig. 2B). In addition, exposure to SMG significantly decreased the ΔΨm of osteoblasts, as indicated by tetramethyl rhodamine methyl ester perchlorate (TMRM) staining (Fig. 2C). These findings suggest that osteoblasts undergo oxidative stress following exposure to SMG. Given the primary role of mitochondrial respiratory chain complexes in cellular oxidative stress, dysfunction of these complexes can result in an increase in ROS production [12]. In this context, we assessed mitochondrial function by measuring the oxygen consumption rate (OCR) and extracellular acidification rate (ECAR). Our results indicated that exposure to SMG reduced basal respiration, maximal respiration, ATP production, and spare respiratory capacity in osteoblasts (Fig. 2D and E), whereas ECAR levels were increased (Fig. 2F and G). Also, the expression of genes associated with OXPHOS, including *NDUFB8*, *UQCRC2*, *SDHB*, and *ATP5F1B*, was down-regulated in SMG-exposed osteoblasts (Fig. 2H). In contrast, SMG up-regulated genes involved in glycolysis, such as *GLUT1*, *HK2*, *PFKM*, and *LDHA* (Fig. 2I), and increased lactate production (Fig. 2J). These findings suggest a metabolic transition in osteoblasts induced by SMG, shifting from OXPHOS to aerobic glycolysis, which reflects a disruption in mitochondrial respiratory functionality. Considering the lower efficiency of ATP production by glycolysis compared to mitochondrial OXPHOS, a decrease in ATP levels was also detected in SMG-exposed osteoblasts (Fig. 2K). Taken together, our results demonstrate that exposure to SMG increases ROS levels and impairs mitochondrial function in osteoblasts, highlighting the susceptibility of osteoblasts to exposure to microgravity environment and the potential impact on osteoblast metabolism.

### Inhibition of mitochondrial fission restores SMG-induced osteoblasts dysfunction

**Fig. 3.**
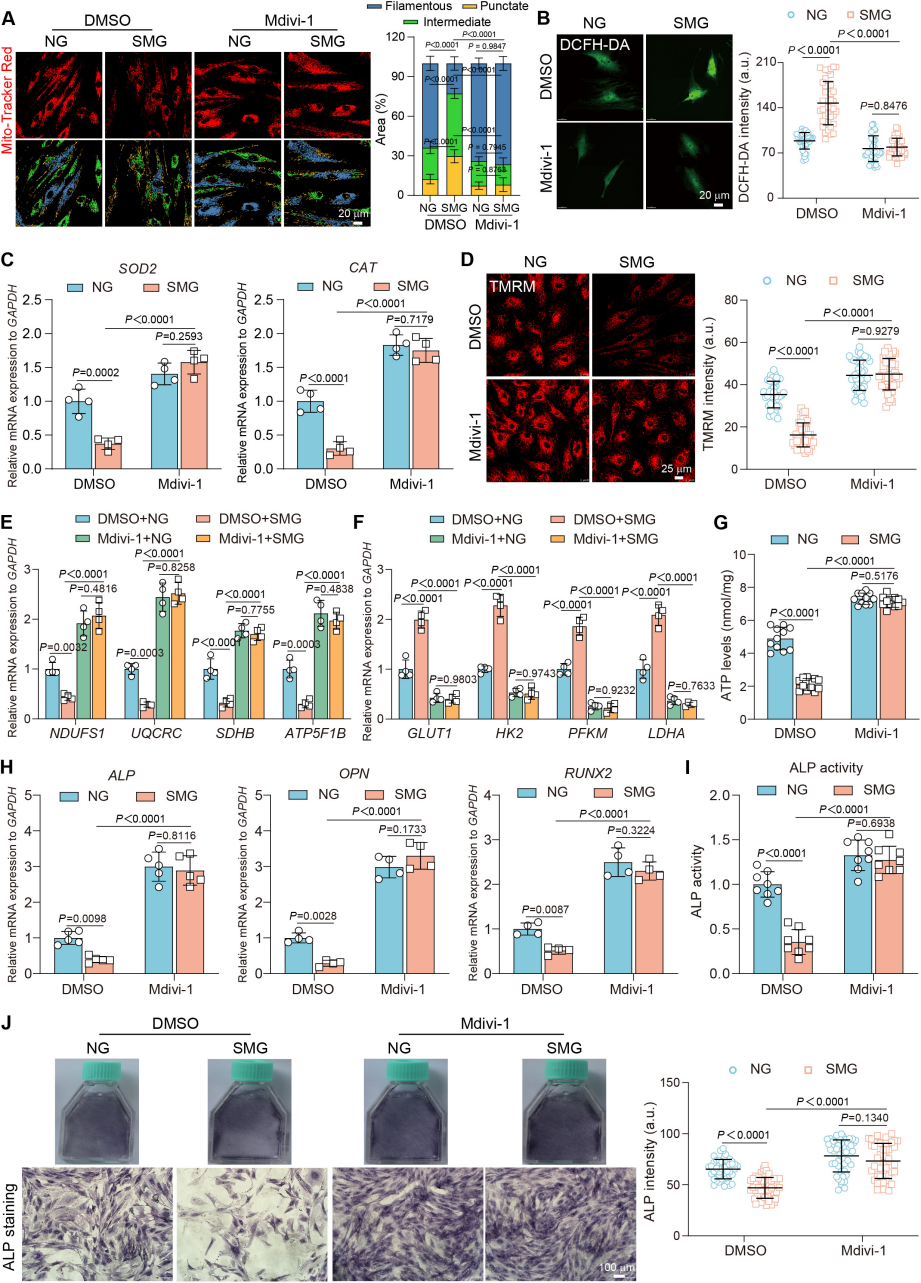
Inhibition of mitochondrial fission restores SMG-induced osteoblast dysfunction. Osteoblasts subjected to treatment with mdivi-1 (25 μM) after 3 days of NG or SMG exposure. (A) Representative immunofluorescent images depicting osteoblast mitochondria labeled with MitoTracker Red, categorized by mitochondrial morphology and displayed with various colors under NG or SMG conditions; scale bars: 20 μm. Classification of mitochondria based on the area percentages of punctate, intermediate, and filamentous structures (*n* = 24 for each group). (B) DCFH-DA staining for ROS; scale bars: 20 μm. Quantification of ROS intensity (*n* = 41 for each group). (C) Gene expression of *SOD2* and *CAT* (*n* ≥ 3). (D) TMRM staining for ΔΨm; scale bars: 25 μm. Quantification of ΔΨm intensity (*n* = 41 for each group). (E and F) Gene expression of oxidative phosphorylation family genes (*NDUFB8*, *UQCRC2*, *SDHB*, and *ATP5F1B*) and glycolysis family genes (*GLUT1*, *HK2*, *PFKM*, and *LDHA*) in osteoblasts (*n* ≥ 3). (G) ATP levels (*n* = 11 for each group). (H) Gene expression of *ALP*, *OPN*, and *RUNX2* (*n* ≥ 3). (I and J) ALP activity (*n* = 8 for each group) and ALP staining and intensity (*n* = 50 for each group); scale bars: 100 μm. Results reflect ≥3 biological repeats, mean ± SD, *P* values via ANOVA or *t* test.

We used the Drp1 inhibitor mdivi-1 to evaluate its protective role against SMG-induced mitochondrial dysfunction in osteoblasts. We assessed both efficacy and safety using a broad range of mdivi-1 concentrations (5, 10, 25, 35, and 50 μM) [31]. Our results confirm that while 50 μM of mdivi-1 significantly reduces osteoblast viability, concentrations from 5 to 35 μM do not adversely affect cell viability, providing a safe concentration range for subsequent studies (Fig. S2). We further evaluated the effect of mdivi-1 on mitochondrial dynamics at concentrations from 5 to 35 μM under SMG conditions. Notably, concentrations of 25 and 35 μM effectively mitigated SMG-induced mitochondrial fission, evidenced by the decreased area of punctate and intermediate mitochondria and increased area of filamentous forms. However, lower concentrations (5 and 10 μM) did not have significant effects, indicating their limited efficacy (Fig. 3A and Fig. S3). Additionally, STED super-resolution microscopy with TOM20 immunolabeling showed that 25 μM of mdivi-1 significantly restored the mitochondrial length and footprint reduction caused by SMG (Fig. S4A and B). TEM further demonstrated that 25 μM of mdivi-1 effectively reversed the increased sphericity and restored the decreased cristae number in mitochondria of osteoblasts exposed to SMG (Fig. S4C and D). Based on these results, we determined that 25 μM of mdivi-1 optimally balances efficacy and safety, providing robust protection against SMG-induced mitochondrial fission while maintaining osteoblast viability. Moreover, our analysis revealed a significant decrease in ROS production and an up-regulation in the gene expression of antioxidant enzymes, such as *SOD2* and *CAT* (Fig. 3B and C), suggesting a reduction in oxidative stress. Mdivi-1 also restored ΔΨm and abolished changes in mitochondrial respiration by down-regulating genes associated with glycolysis and up-regulating those involved in OXPHOS (Fig. 3D to F). These adjustments led to the restoration of ATP production that were affected by exposure to SMG (Fig. 3G). Further extending our analysis to osteoblast function, we found that mdivi-1 induced a marked recovery in the expression of essential osteogenic markers, namely, *ALP*, *OPN*, and *RUNX2*, in osteoblasts exposed to SMG for 3 days (Fig. 3H). This recovery was accompanied by an enhancement in ALP activity and staining intensity (Fig. 3I and J). Considering the crucial role of collagen in osteoblast function, we also observed a significant reduction in collagen I expression under SMG conditions, which was effectively reversed by mdivi-1 (Fig. S5A and B). We also noted a substantial decrease in hydroxyproline concentration, a specific amino acid in collagen [32], which was successfully restored by treatment with mdivi-1, further affirming its efficacy to alleviate osteoblast dysfunction caused by SMG (Fig. S5C). To strengthen the robustness of our pharmacological findings related to mdivi-1, we conducted further experiments focusing on the genetic regulation of Drp1 expression. Drp1 knockdown not only maintained mitochondrial morphology and decreased SMG-induced mitochondrial fission but also reduced oxidative stress while increasing ΔΨm and respiration (Fig. S6A to G). This facilitated the recovery of ATP production and improved osteoblast function previously compromised by exposure to SMG (Fig. S6H to K). The Drp1 knockdown replicated the protective effects of the mdivi-1 treatment, underscoring the critical role of Drp1 in mediating SMG-induced mitochondrial dysfunctions. Overall, our findings emphasize the effectiveness of blocking mitochondrial fission in overcoming SMG-induced osteoblast impairment, highlighted by ROS suppression and mitochondrial function improvement.

### Disruption of mechanotransduction impairs osteoblast function under SMG conditions

**Fig. 4.**
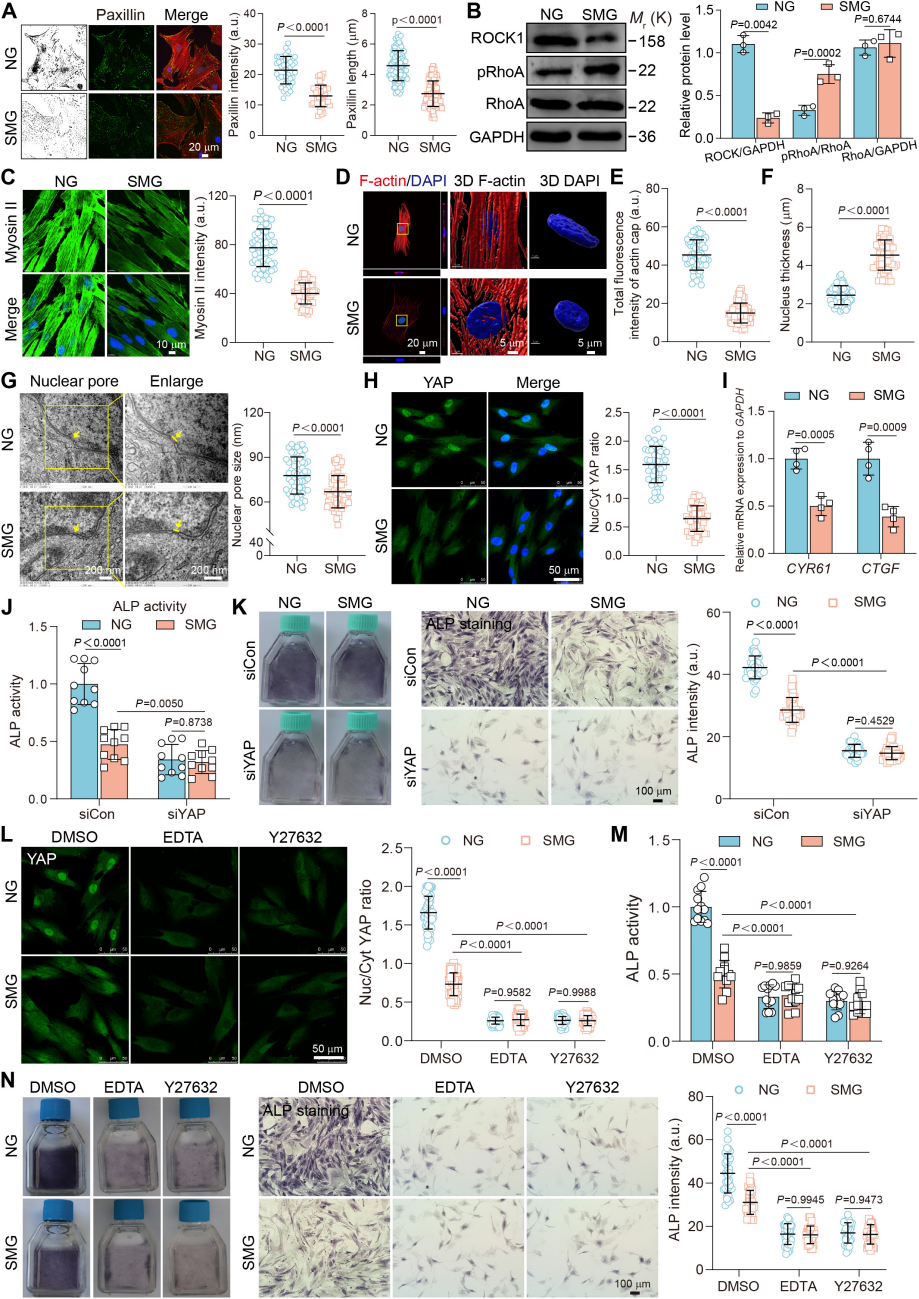
Disruption of mechanotransduction impairs osteoblast function under SMG conditions. (A) F-actin (red) and paxillin (green) immunostaining after 3 days of NG or SMG exposure; scale bars: 20 μm. Paxillin intensity (*n* = 44 for each group) and length (*n* = 126 for each group) quantifications. (B) The representative images and quantitative analysis of the protein level expression of RhoA (phospho S188), RhoA, and ROCK1 after 3 days of NG or SMG exposure (*n* = 3). (C) Immunostaining for Myosin II after 3 days of NG or SMG exposure; scale bars: 10 μm. Quantification of Myosin II intensity (*n* = 61 for each group). (D) 3D rendering indicated actin cap and nuclear morphology after 3 days of NG or SMG exposure. Yellow boxes indicate the regions that were shown as a magnified view; scale bars: 20 μm and 5 μm. (E) Total fluorescence intensity of actin cap quantifications (*n* = 62 for each group). (F) Nucleus thickness metrics (*n* = 62 for each group). (G) TEM images showing nuclear pores after 3 days of NG or SMG exposure. Yellow boxes indicate the regions that were shown as a magnified view. Analysis of nuclear pore size (*n* = 58 for each group from ≥10 cells). Scale bars: 200 nm. (H) YAP localization immunostaining after 3 days of NG or SMG exposure; scale bars: 50 μm. YAP nuclear–cytoplasmic intensity ratio quantifications (*n* = 57 for each group). (I) The gene expression of *CYR61* and *CTGF* after 3 days of NG or SMG exposure (*n* ≥ 3). (J and K) Osteoblasts transfected with siCon or siYAP after 3 days of NG or SMG exposure, followed by an ALP activity assay (*n* = 10), ALP staining, and ALP intensity (*n* = 47 for each group); scale bars: 100 μm. Osteoblasts subjected to treatment with chaetocin (5 μM) or GSK343 (5 μM) after 3 days of NG or SMG exposure, followed by an ALP activity assay (*n* = 13). (L) Osteoblasts subjected to treatment with EDTA (1.25 mM) or Y27632 (10 μM) after 3 days of NG or SMG exposure. YAP localization immunostaining; scale bars: 50 μm. YAP nuclear–cytoplasmic intensity ratio (*n* = 48 for each group). (M and N). Osteoblasts subjected to treatment with EDTA (1.25 mM) or Y27632 (10 μM) after 3 days of NG or SMG exposure, followed by an ALP activity assay (*n* = 10), ALP staining, and ALP intensity (*n* = 47 for each group); scale bars: 100 μm. Results reflect ≥3 biological repeats, mean ± SD, *P* values via ANOVA or *t* test.

Our study also investigated the effects of exposure to SMG on the mechanotransduction ability of osteoblasts, focusing on the FA–cytoskeleton–nucleus axis. We found a significant decrease in both the staining intensity and length of paxillin (Fig. 4A), a critical component of the FA complex crucial for the transmission of mechanical forces from the ECM to the actin cytoskeleton [33]. Additionally, our study revealed a suppression of the RhoA–ROCK–Myosin II pathway, which plays a pivotal role in the sensing of mechanical stimuli [34]. This suppression was supported by results showing increased phosphorylation of the inactive form of RhoA, a decrease in ROCK1 expression, and unchanged levels of RhoA (Fig. 4B), coupled with a reduction in Myosin II expression (Fig. 4C). Notably, we also detected a decrease in filamentous actin (F-actin) and an increase in globular actin (G-actin) (Fig. S7A and B), alongside reduced formation of the perinuclear actin cap (Fig. 4D and E). These alterations indicate an impaired ability of osteoblasts to sense and transmit mechanical signals, thereby disrupting the function of the mechanosensitive machinery, including paxillin, RhoA, ROCK1, Myosin II, and the actin cap, all of which are essential for maintaining nuclear shape and architecture. Under SMG conditions, we further observed significant changes in nuclear architecture, characterized by an increase in nucleus thickness and a reduction in nuclear area and volume (Fig. 4F and Fig. S8), as well as a contraction of nuclear pores (Fig. 4G). We also investigated the role of the mechanotransducer YAP, which was released from the nucleus under SMG conditions (Fig. 4H), leading to the down-regulation of its target genes, *CTGF* and *CYR61* (Fig. 4I). Furthermore, YAP knockdown resulted in decreased ALP activity and staining intensity in both normal gravity (NG) and SMG conditions. Notably, this knockdown exacerbated the detrimental effects of SMG on osteoblast function compared to the control (siCon), as evidenced by a greater reduction in ALP activity and staining intensity following siYAP treatment (Fig. 4J and K and Fig. S9). This underscores the critical role of YAP in SMG-induced osteoblast dysfunction. Additionally, the use of pharmacological inhibitors (specifically EDTA and Y27632 to inhibit the FA complex and ROCK, respectively) led to reduced nuclear localization of YAP in both NG and SMG conditions. This reduction was more pronounced under SMG conditions compared to the control (dimethyl sulfoxide [DMSO]), highlighting the crucial role of mechanosensitive components in modulating localization of YAP response under SMG conditions (Fig. 4L). Moreover, treatment with these inhibitors significantly exacerbated SMG-induced osteoblast dysfunction, evidenced by a greater reduction in ALP activity and staining intensity compared to DMSO (Fig. 4M and N). Our findings demonstrate that exposure to SMG significantly disrupts the mechanotransduction pathways in osteoblasts, impairing their structural integrity and functional capacity for osteogenic differentiation, stressing the indispensable roles of mechanosensitive elements in cellular adaptation to mechanical environments.

### Alleviating SMG-induced osteoblast dysfunction by targeting epigenetic modifications

**Fig. 5.**
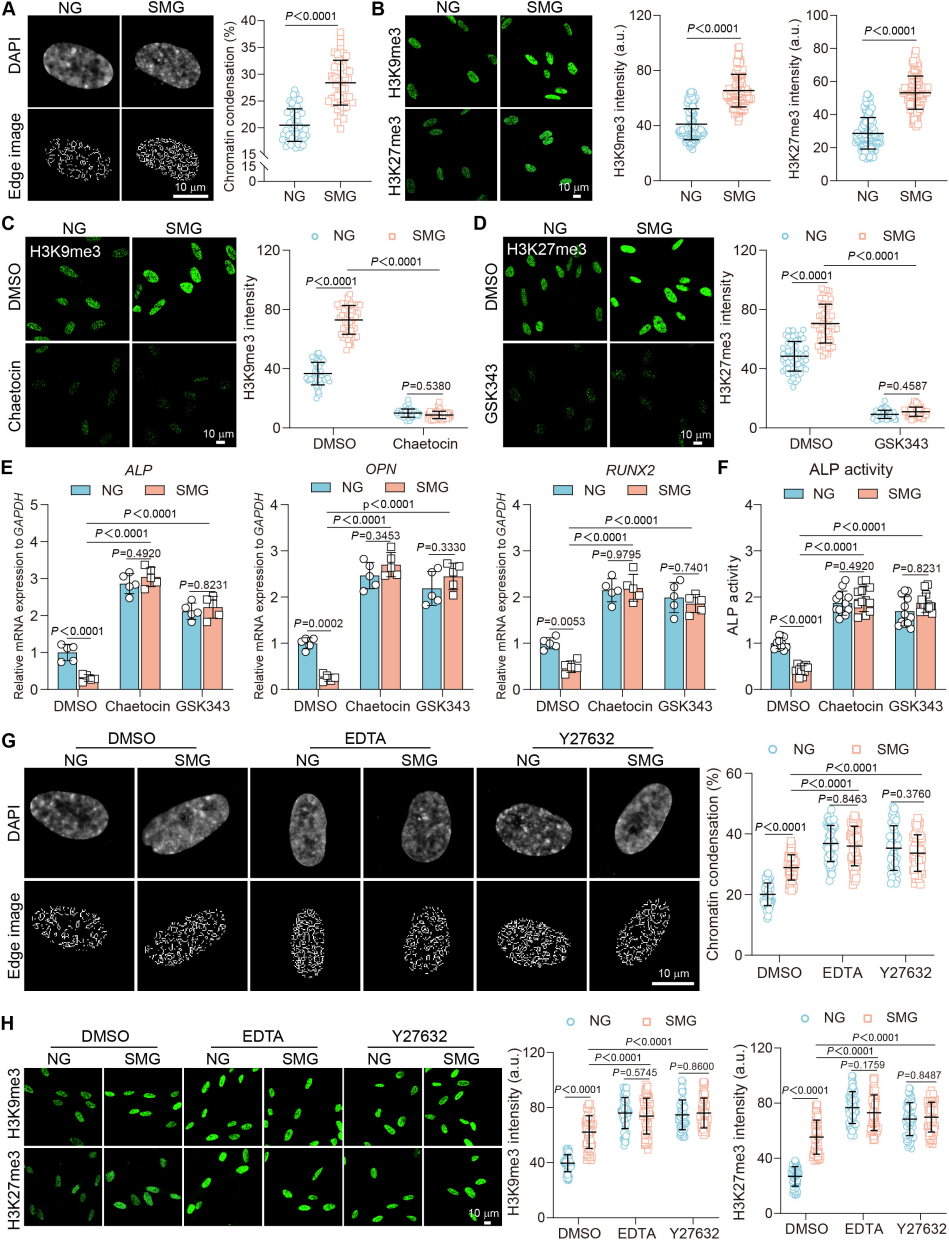
Alleviating SMG-induced osteoblast dysfunction by targeting epigenetic modifications. (A) Nucleus DAPI staining and CCP edge detection after 3 days of NG or SMG exposure; scale bars: 10 μm. CCP quantification (*n* = 55 for each group). (B) H3K9me3 and H3K27me3 immunostaining after 3 days of NG or SMG exposure; scale bars: 10 μm. H3K9me3 (*n* = 112 for each group) and H3K27me3 (*n* = 127 for each group) intensity quantifications. (C) Osteoblasts treated with the DMSO or H3K9me3 inhibitor chaetocin (5 μM) under NG or SMG conditions. H3K9me3 staining and intensity quantifications (*n* = 55 for each group); scale bars: 10 μm. (D) Osteoblasts treated with the DMSO or H3K27me3 inhibitor GSK343 (5 μM) under NG or SMG conditions. H3K27me3 staining and intensity quantifications (*n* = 61 for each group); scale bars: 10 μm. (E and F) Osteoblasts subjected to treatment with chaetocin (5 μM) or GSK343 (5 μM) after 3 days of NG or SMG exposure. Gene expression of *ALP*, *OPN*, and *RUNX2* (*n* ≥ 3) and ALP activity (*n* = 8 for each group). (G) Osteoblasts subjected to treatment with EDTA (1.25 mM) or Y27632 (10 μM) after 3 days of NG or SMG exposure. Nucleus DAPI staining and CCP edge detection; scale bars: 10 μm. CCP quantification (*n* = 52 for each group). (H) H3K9me3 and H3K27me3 immunostaining; scale bars: 10 μm. H3K9me3 (*n* = 65 for each group) and H3K27me3 (*n* = 64 for each group) intensity quantifications. Results reflect ≥3 biological repeats, mean ± SD, *P* values via ANOVA or *t* test.

Epigenetic remodeling plays a crucial role in osteogenic differentiation, modulated by biomechanical signals through alterations in nuclear architecture and leading to substantial epigenetic modifications [35]. Our study found that SMG conditions markedly increased the chromatin condensation parameter (CCP) (Fig. 5A), indicating a shift toward transcriptional silencing, and elevated the levels of H3K9me3 and H3K27me3 (Fig. 5B), which are markers of heterochromatin and major epigenetic barriers to differentiation. The use of 2 specific inhibitors, namely, chaetocin and GSK343, targeting the H3K9 methyltransferase Su(var)3–9 and the H3K27 methyltransferase EZH2, respectively, effectively counteracted the SMG-induced increases in H3K9me3 and H3K27me3 levels (Fig. 5C and D). These inhibitors also prevented the SMG-mediated down-regulation of the mRNA levels of critical osteogenic markers, including *ALP*, *OPN*, and *RUNX2* (Fig. 5E), as well as the ALP activity (Fig. 5F), underscoring their significance as epigenetic barriers to osteogenic differentiation. Furthermore, the treatment with EDTA or Y27632 intensified the effects of SMG on the CCP, H3K9me3, and H3K27me3 levels (Fig. 5G and H), highlighting the critical role of mechanosensitive elements in modulating chromatin structure and epigenetic responses to SMG. Collectively, these findings suggest a complex interaction between SMG and the mechanosensitive machinery along the cytoskeleton–nuclear axis, which regulates epigenetic modifications and thus controls the SMG-induced osteoblast dysfunction.

### Inhibition of mitochondrial fission reverses SMG-induced disruptions in mechanotransduction and epigenetic modification

**Fig. 6.**
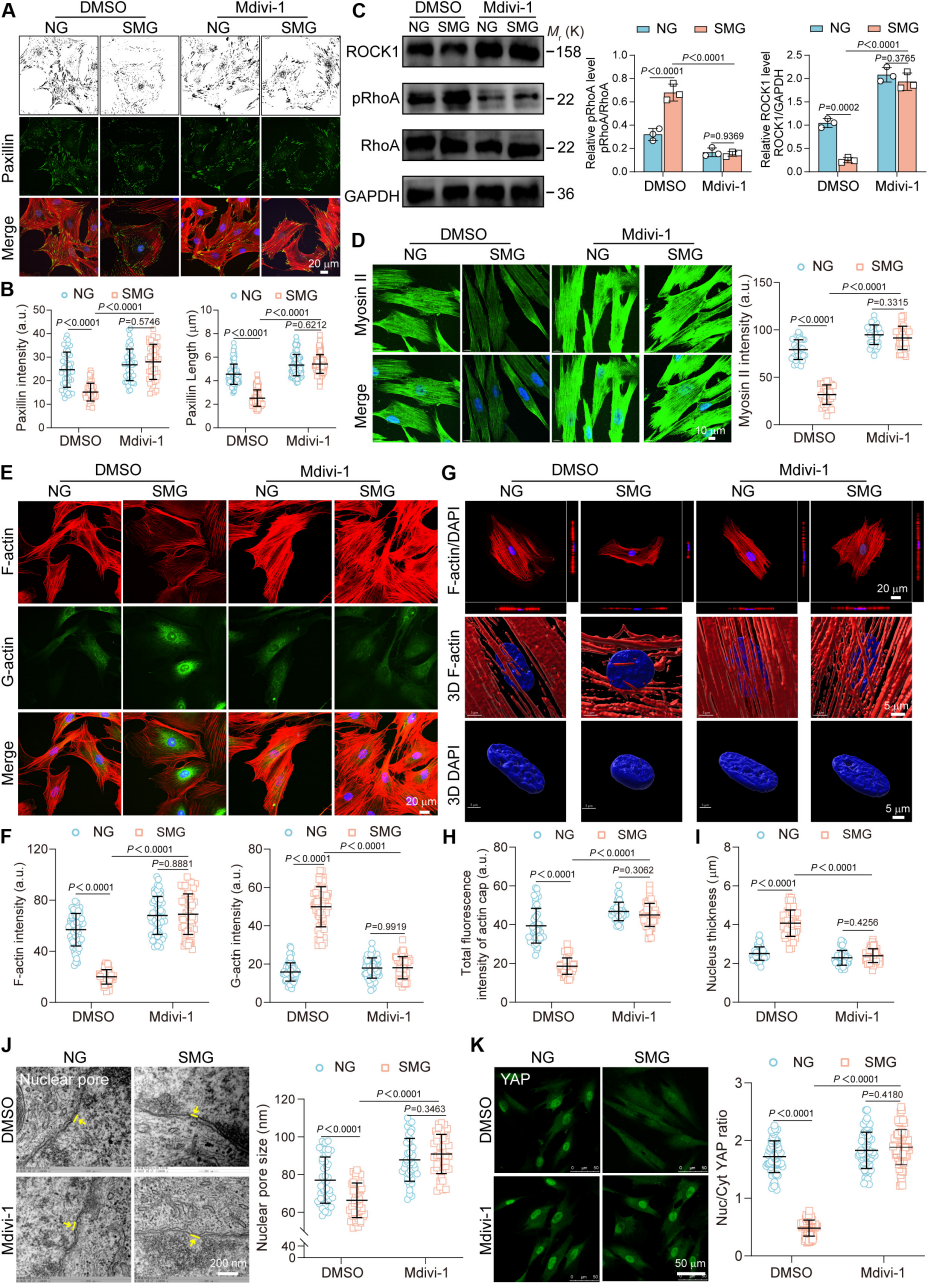
Inhibition of mitochondrial fission reverses SMG-induced disruptions in mechanotransduction. Osteoblasts subjected to treatment with mdivi-1 (25 μM) after 3 days of NG or SMG exposure. (A) F-actin (red) and paxillin (green) immunostaining; scale bars: 20 μm. (B) Paxillin intensity (*n* = 48 for each group) and length (*n* = 118 for each group) quantifications. (C) The representative images and quantitative analysis of the protein level expression of RhoA (phospho S188), RhoA, and ROCK1 (*n* = 3). (D) Myosin II immunostaining; scale bars: 10 μm. Myosin II intensity metrics (*n* = 36 for each group). (E) F-actin (red) and DNase I 488 for G-actin (green) immunostaining; scale bars: 20 μm. (F) F-actin and G-actin intensity quantifications (*n* = 59 for each group). (G) 3D rendering indicated actin cap and nuclear morphology. Yellow boxes indicate the regions that were shown as a magnified view; scale bars: 20 and 5 μm. (H) Total fluorescence intensity of actin cap (*n* = 48 for each group). (I) Nucleus thickness assessed (*n* = 48 for each group). (J) TEM of nuclear pores. Yellow boxes indicate the regions that were shown as a magnified view; scale bars: 200 nm. Nuclear pore size analysis (*n* = 43 for each group from ≥ 12 cells). (K) YAP immunostaining; scale bars: 50 μm. YAP nuclear–cytoplasmic intensity ratio (*n* = 67 for each group). Results reflect ≥3 biological repeats, mean ± SD, *P* values via ANOVA or *t* test.

**Fig. 7.**
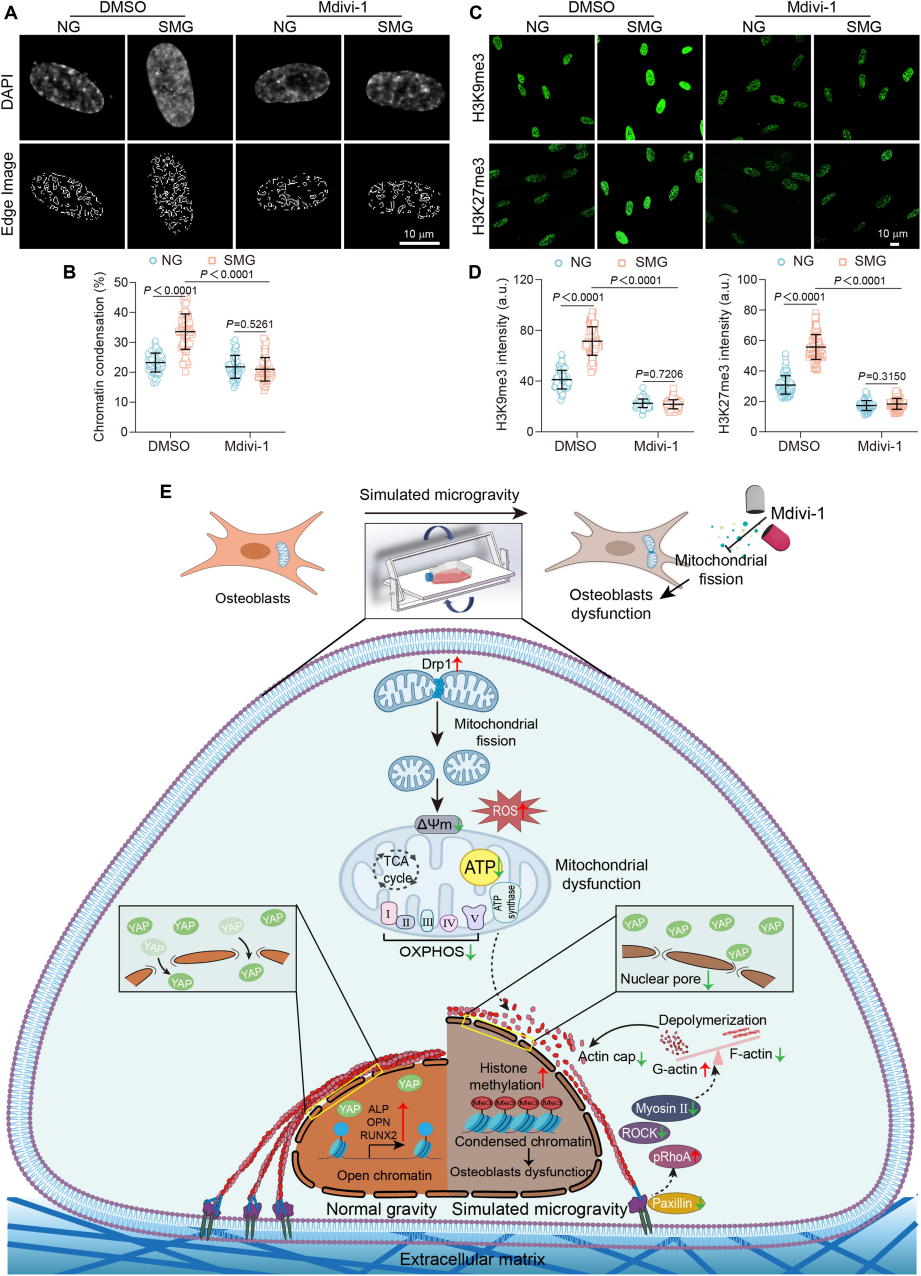
Inhibiting mitochondrial fission reverses SMG-induced epigenetic modification. Osteoblasts subjected to treatment with mdivi-1 (25 μM) after 3 days of NG or SMG exposure. (A) DAPI staining and CCP edge detection; scale bars: 10 μm. (B) CCP quantification (*n* = 62 for each group). (C) H3K9me3 and H3K27me3 immunostaining; scale bars: 10 μm. (D) H3K9me3 (*n* = 80 for each group) and H3K27me3 (*n* = 107 for each group) intensity quantifications. (E) The schematic provides a detailed depiction of how inhibiting mitochondrial fission influences mechanotransduction pathways and epigenetic modification, effectively counteracting osteoblast dysfunction triggered by SMG. Results reflect ≥3 biological repeats, mean ± SD, *P* values via ANOVA or *t* test.

To further investigate the role of mitochondrial fission in mechanotransduction and epigenetic modification, we examined the effect of the fission inhibitor mdivi-1 on these processes. Mdivi-1 maintained the integrity of FAs, as revealed by the restored staining intensity and length of paxillin under SMG conditions (Fig. 6A and B). This preservation underscores a direct connection between mitochondrial dynamics and the mechanical stability of osteoblasts. Furthermore, mdivi-1 effectively restored the SMG-induced disruptions in the RhoA-ROCK signaling pathway, as indicated by the decreased phosphorylation of inactive RhoA and increased ROCK1 expression (Fig. 6C), indicating the recovery of normal signaling functions. This was accompanied by a consequential recovery in Myosin II expression after 3 days of exposure to SMG (Fig. 6D). The treatment effectively maintained the integrity of the cytoskeletal structure under SMG conditions, as revealed by an increase in F-actin and a decrease in G-actin, thus preserving the cytoskeletal architecture (Fig. 6E and F). The re-establishment of actin cap structures, which are vital for the transmission of mechanical forces to the nucleus, was also achieved by treatment with mdivi-1 (Fig. 6G and H). Moreover, mdivi-1 reversed the SMG-induced changes in nuclear morphology, as indicated by a flatter nuclear shape and increased nuclear area, volume, and pore dimensions (Fig. 6I and J and Fig. S10). These changes suggest a reconstitution of the SMG-disrupted nuclear architecture. Additionally, the inhibitor counteracted the SMG-induced nuclear release of YAP (Fig. 6K), restoring the suppressed expression levels of *CTGF* and *CYR61* (Fig. S11). These results underscore the pivotal role of mitochondrial fission in maintaining the integrity of mechanosensitive signaling pathways in osteoblasts under SMG conditions. Importantly, mdivi-1 mitigated the SMG-induced increase in the CCP and effectively prevented the up-regulation of H3K9me3 and H3K27me3 (Fig. 7A to D). Given the crucial role of H3K9 and H3K27 methylation as epigenetic barriers to osteoblast function under SMG conditions, our study supports the attenuation of these epigenetic markers through the inhibition of mitochondrial fission as a therapeutic approach. By reducing these methylation marks, the inhibition of mitochondrial fission may facilitate chromatin relaxation, thus providing a viable strategy to alleviate SMG-induced osteoblast dysfunction.

Overall, our study reveals that exposure to SMG leads to the suppression of osteogenic markers and induces mitochondrial fission, resulting in increased oxidative stress and a metabolic shift toward glycolysis, which, in turn, impairs ATP production, indicating mitochondrial dysfunction. The treatment with mdivi-1 not only maintains mitochondrial structure and reduces ROS production, but also rescues mitochondrial function, thereby restoring osteoblast differentiation. Furthermore, this treatment prevents SMG-induced disruption in mechanotransduction pathways and nuclear architecture. Epigenetic analysis found SMG-induced chromatin remodeling, characterized by increased H3K9me3 and H3K27me3 levels, which were reduced by mdivi-1 treatment. Overall, our findings identify mitochondrial fission as a key mechanism influencing the osteoblast response to SMG, providing a promising target for therapeutic intervention to safeguard skeletal health in a microgravity environment (Fig. 7E).

## Discussion

This study elucidated the complex interplay between mitochondrial dynamics, oxidative stress, mechanotransduction, and epigenetic modifications in osteoblasts under SMG conditions, providing crucial insights into the cellular and molecular mechanisms underlying bone loss in space. These findings form a foundational basis for developing targeted interventions aimed at overcoming this major obstacle to long-term space exploration and astronaut health.

Mitochondrial dynamics are critical to cellular energy metabolism and pivotal for the functionality of osteoblasts, the cells responsible for bone formation and remodeling [36]. Our study revealed a significant shift toward enhanced mitochondrial fission in osteoblasts when exposed to SMG, characterized by an increase in mitochondrial fragmentation and a reduction in crista density. These cristae, folds in the inner mitochondrial membrane, house the electron transport chain (ETC) complexes essential for ATP production [37]. The association between mitochondrial fission and reduced ATP production under SMG conditions is further supported by our observations of decreased ΔΨm. The membrane potential is a driving force for ATP synthesis, generated by the ETC as it pumps protons across the inner mitochondrial membrane [38]. A decrease in ΔΨm implies that the capacity of the ETC to synthesize ATP is compromised, leading to a potential energy crisis within the cell [39]. In response to this bioenergetic challenge, cells may shift their metabolic strategy from OXPHOS, which is highly efficient but dependent on a functional ETC, to glycolysis, which is less efficient but can occur independently of mitochondrial integrity [40]. This shift, while ensuring a degree of energetic survival, comes at the cost of significantly reduced ATP yield per molecule of glucose consumed [41]. For osteoblasts, which are highly energy-dependent due to their role in bone formation and remodeling [42], this metabolic shift can result in insufficient ATP to sustain normal function, leading to the observed down-regulation of osteogenic markers and impaired differentiation. Noteworthy, we found that mdivi-1, a mitochondrial fission inhibitor, holds therapeutic promise in preventing the detrimental effects of SMG on osteoblasts. The results in a 3-dimensional (3D) GelMA hydrogel culture system were consistent with those from 2-dimensional (2D) studies (Figs. S12A to E and S13A to C). This treatment not only preserved mitochondrial morphology but also enhanced osteoblast differentiation and function.

Mechanotransduction plays a critical role in osteoblast function and overall bone health, serving as an important mechanism by which cells perceive and respond to mechanical cues [43]. Previous research has highlighted the crucial roles played by mechanosensitive molecules, such as FAs, F-actin, and YAP, in sustaining osteoblast activity [44]. Our study corroborates these insights, revealing that SMG compromises key components of the osteoblast mechanotransduction machinery. We documented the compromised integrity of FAs, perturbed RhoA-ROCK signaling pathway activity, and disorganization within the actin cytoskeleton. Such disruptions not only alter nuclear architecture but also displace YAP from its regulatory domain, impairing the transcriptional control of genes essential for bone development [45]. This observation emphasizes the necessity for treatments that enhance mechanical signaling mechanisms in osteoblasts under SMG conditions. Moreover, mechanotransduction processes, including the formation of FAs and the polymerization of the actin cytoskeleton, are energetically demanding [46,47]. Our findings suggest that SMG-induced mitochondrial fission contributes to a reduction in ATP production, thus depleting the energy reservoir required for these critical mechanotransduction functions. Previous research has revealed the importance of cellular energy in regulating nuclear attributes, such as nuclear envelope tension and nuclear pore size [48]. Our findings extend this finding by showing that exposure to SMG prompts significant changes in the nucleus, affecting nuclear pore dimensions and misaligning critical transcriptional regulators like YAP. These changes also indicate the wide-reaching consequences of the energy deficit, impairing the mechanosensing ability of osteoblasts [49]. The treatment with mdivi-1 has emerged as an effective strategy to successfully restore mitochondrial integrity and ATP production. This treatment significantly improves cellular mechanics under SMG conditions, strengthening mechanosensing and signal transduction processes. Mdivi-1 facilitates the reconstruction of FAs, actin polymerization, and nuclear stability, offering a strategic approach to mitigate the adverse effects of microgravity on skeletal health.

Besides functioning as a vault for genetic material, the nucleus plays a crucial role in converting mechanical signals into biochemical responses [50]. This study elucidated the impacts of an energy deficit, precipitated by mitochondrial fission, on nuclear architecture. The actin cytoskeleton, which is dependent on ATP for its function, maintains nuclear shape by exerting tension on the nuclear lamina and envelope [51,52]. Our findings revealed that decreased ATP levels lead to a reduction in this tension, culminating in notable changes in nuclear morphology, including variations in thickness, area, and volume. These morphological changes can significantly influence gene expression by altering the organization of chromatin within the nucleus [53,54]. Usually, more condensed chromatin is less accessible to transcriptional machinery, potentially resulting in a widespread down-regulation of gene expression [55]. Our findings indicate that SMG markedly induces chromatin condensation. Additionally, epigenetic modifications, such as histone methylation, play a role in either tightening or loosening chromatin structure, thus influencing gene accessibility [56]. Our study demonstrates that SMG significantly increases the levels of histone methylation markers (H3K9me3 and H3K27me3), indicating a repressed chromatin state. The increase in these methylation markers under SMG conditions could imply a cellular strategy to conserve energy by broadly reducing transcriptional activity as a response to the energy shortage [57]. By restoring ATP levels, mdivi-1 has the potential to reverse the alterations in the chromatin state and methylation patterns, thereby restoring osteoblast function.

Our study provides a comprehensive analysis of the effects of SMG on osteoblasts, focusing on mitochondrial dynamics, mechanotransduction, and epigenetic modifications. Using mdivi-1 to inhibit mitochondrial fission, we uncovered new therapeutic approaches to counteract SMG-induced cellular dysfunctions. This strategy enables a holistic understanding of the complex relationships among mitochondrial dysfunction, mechanotransduction impairment, and epigenetic changes, thereby providing an integrated view of osteoblast responses to microgravity. Introducing mdivi-1 as a novel countermeasure represents a substantial advancement in space medicine, aiming to alleviate the detrimental effects of microgravity on cellular functions. However, the validity of our findings is tempered by the limitations of using a 2D rotating apparatus, which might not fully replicate the complexities of the space environment, and the lack of long-term data on the effects of mdivi-1, which is crucial for assessing its efficacy for extended space missions. Our study employed rat osteoblasts instead of human osteoblasts, which may limit the direct applicability of our findings to human physiology. Moreover, our research focused primarily on the molecular mechanisms within osteoblasts and did not address interactions with other crucial components of the bone microenvironment, such as mesenchymal stem cells and osteoclasts. These interactions are essential for a comprehensive understanding of bone remodeling. Despite these challenges, our research provides valuable insights into strategies for maintaining bone density in microgravity environment, potentially improving astronaut health on prolonged spaceflights and advancing osteoporosis treatments on Earth.

In summary, this study describes a link between SMG conditions and osteoblast dysfunction through enhanced mitochondrial fission, unveiling a cascade of intracellular changes from mitochondrial morphological alterations and increased oxidative stress, to the disruption of mechanotransduction pathways. Our findings not only reveal the pivotal role of mitochondrial dynamics in mediating osteoblast response to SMG, but also show the novel therapeutic potential of targeting mitochondrial fission to alleviate this dysfunction. By using the mitochondrial fission inhibitor mdivi-1, we demonstrated an innovative approach to maintaining mitochondrial structure, reducing oxidative stress, and restoring both mechanotransduction and epigenetic modifications under SMG conditions. This strategic intervention not only restores osteoblast differentiation but also opens new avenues for safeguarding skeletal health in microgravity environment, potentially providing a novel countermeasure against bone loss during long-duration space exploration.

## Materials and Methods

### Primary rat osteoblasts isolation and culture

In accordance with the protocols delineated in our prior publication [9], primary osteoblasts were extracted from the calvariae of neonatal Sprague–Dawley rats, sourced within 24 h post-birth from Peking University Third Hospital, Beijing, China. This research was conducted in strict adherence to the ethical standards of the Declaration of Helsinki and received approval from the Beihang University Ethics Committee. The extraction of osteoblasts was initiated by aseptically dissecting the calvariae and scrupulously excising all attached soft tissue. Enzymatic dissociation of the bone fragments was performed at 37 °C, first with 0.25% trypsin (Sigma-Aldrich, Germany) for 2 15-min periods and subsequently with 3 mg/ml collagenase I (Sigma-Aldrich, Germany) for 5 10-min periods. Following digestion, the cells were collected, passed through a 75-μm cell strainer, centrifuged, and then suspended in Dulbecco’s modified Eagle’s medium (DMEM; Gibco, USA) supplemented with 10% heat-inactivated fetal bovine serum (FBS; Gibco, USA). The cells were cultured in a humidified 5% CO_2_ atmosphere at 37 °C to confluence. Passage of the osteoblasts was achieved using 0.25% trypsin. Experiments were conducted using cells from the third to the sixth passages. Stability of the osteogenic phenotype in osteoblasts at passages P1, P3, and P6 was confirmed via ALP staining, ALP activity assays, and Alizarin Red S staining (Fig. S14A to C).

### Simulated microgravity

The 2D clinostat, which alters cell orientation through continuous rotation, was used to SMG for in vitro culture, as detailed in our previous study [9]. This method operates on the principle that when cells affixed to a slide rotate 90°, the gravitational vector shifts, averaging to nearly 0 *g* over a full 360° rotation, rendering gravity undetectable to cells (Fig. S15). Osteoblasts were seeded at 4,000 cells/cm^2^ in culture flasks (12.5 cm^2^, Becton Dickinson, USA) and allowed 24 h for adhesion. Afterward, the medium was refreshed with DMEM and 10% FBS, ensuring air bubbles were removed to reduce fluid shear stress. In the SMG group, flasks attached to the 2D clinostat rotated at 15 rpm, simulating a gravitational force of about 10^−3^ g [9,58]. The setup resided in an incubator with a humidified atmosphere of 5% CO_2_ at 37 °C. Control samples were cultured under NG without clinorotation.

### Quantitative real-time PCR

Total RNA was extracted utilizing TRIzol reagent (Invitrogen, USA) according to the manufacturer’s specifications. The concentration of RNA was quantified using an ND-1000 spectrophotometer. Following this, 2 μg of RNA was reverse-transcribed into cDNA. Quantitative real-time PCR (qRT-PCR) was then conducted on an Applied Biosystems 7900HT Fast Real-Time PCR system using specific primers. Expression data were normalized against the mRNA levels of the housekeeping gene *GAPDH* and analyzed using the 2^−ΔΔCT^ method. Primer sequences are provided in Table S1.

### ALP activity assay and staining

ALP activity in osteoblasts was quantitatively assessed using a commercial ALP assay kit (#A059-1-1, Nanjing Jiancheng Bioengineering Institute, China) according to the manufacturer’s instructions. For ALP staining, osteoblasts underwent a washing step with PBS followed by fixation with 4% paraformaldehyde (PFA). Subsequent to fixation, cells were stained employing a 5-bromo-4-chloro-3-indolyl phosphate/nitro blue tetrazolium (BCIP/NBT) ALP staining kit (#C3206, Beyotime Biotechnology, China). Post-staining, osteoblasts were visualized and imaged using light microscopy. For quantitative assessments, ImageJ software was utilized to analyze the stained specimens.

### Alizarin red S staining

Calcium nodules generated by the osteoblasts were stained using an Alizarin Red S staining kit (#C0148S, Beyotime Biotechnology, China). Osteoblasts from passages P1, P3, and P6 were cultured for 14 days and stained with Alizarin S Red. After washing with PBS, the osteoblasts were fixed with 4% PFA for 30 min and then stained with Alizarin Red for 15 min. The stained calcium nodules were visualized using a microscope. For quantification, the Alizarin Red stain was dissolved in an alkyl hexadecylpyridine solution and the optical density (OD) at 562 nm was measured using a microplate reader.

### Hydroxyproline assay

Hydroxyproline concentrations were quantified employing a hydroxyproline detection kit as per the manufacturer’s instructions (#D799573-0050, Sangon Biotech, China). Briefly, preparations of cell culture supernatants, deionized water, and a standard protein sample were combined with an equivalent volume of NaOH. This mixture was evaporated, subsequently cooled, and neutralized with a corresponding volume of HCl. The samples were then centrifuged to separate the supernatants, which were transferred to clean tubes. Each sample was treated with an oxidation reagent and allowed to react at room temperature for 20 min. A developer solution was subsequently added, and the samples were incubated at 37 °C for 5 min. This procedure was followed by the addition of DMAB concentrate, and the samples were further incubated at 65 °C for 45 min. The OD of each sample was measured at 560 nm, and hydroxyproline levels were determined using a predefined standard curve.

### MitoTracker Red staining

Osteoblasts were stained with MitoTracker Red (#C1049B, Beyotime Biotechnology, China) for 10 min at 37 °C. Cells were incubated with the dye for 10 min at 37 °C, followed by thorough washing with PBS and a subsequent medium replacement with DMEM. Mitochondrial morphology was visualized using a confocal laser-scanning microscope (Andor Dragonfly 500, Leica, England). The classification of mitochondrial morphology was quantified by a machine-learning as described previously [28]. The mitochondrial morphology was classified as punctate, intermediate, and filamentous, and then calculated the percentage area of each category in each image.

### Transmission electron microscopy

Ultrastructural analysis of mitochondria and nuclear pores in osteoblasts was performed using a Hitachi 7065B TEM (Hitachi, Japan). Osteoblasts were initially harvested with precooled PBS and subsequently fixed in 2.5% glutaraldehyde at 4 °C overnight. After fixation, the cells were rinsed with PBS and further fixed in a solution containing 0.8% potassium ferrocyanide and 2% osmium tetroxide in 0.1 M sodium cacodylate buffer for 1 h. This was followed by a graded dehydration process using increasing concentrations of alcohol, after which specimens were embedded in epoxy resin. Ultrathin sections of 60 nm were cut using a UC7 ultramicrotome (Leica Microsystems, Australia), stained sequentially with 5% uranyl acetate for 10 min and lead citrate for 10 min, and mounted on 200-mesh copper grids. Examination was performed using the TEM at 80 kV, with image capture facilitated by a charge-coupled device camera (#SC1000, Gatan, USA). Mitochondrial morphology was quantitatively analyzed using ImageJ software, with the globularity index of mitochondria calculated using the formula (min/max)^2^. Nuclear pores were characterized by their distinct nuclear baskets, and pore length was measured from one side of the nuclear double bilayer at the commencement of the nuclear basket to the opposing side at the termination of the nuclear basket.

### Western blot assay

Cell lysis was conducted using precooled PBS followed by RIPA buffer (Beyotime Biotechnology, China) supplemented with phenylmethylsulfonyl fluoride (Beyotime Biotechnology, China) and a protease inhibitor cocktail (Beyotime Biotechnology). For Western blot analysis, proteins, in amounts ranging from 30 to 40 μg per sample, were resolved via SDS-PAGE and then transferred onto polyvinylidene fluoride membranes. These membranes were blocked with 5% bovine serum albumin (BSA) in Tris-buffered saline containing 0.1% Tween-20 for 2 h at room temperature. Primary antibodies were applied for overnight incubation at 4 °C, including anti-Drp1 (1:800, #ab56788, Abcam), anti-Mfn1 (1:500, #ab57602, Abcam), anti-Mfn2 (1:500, #ab56889, Abcam), anti-Collagen I (1:1,000, #ab6308, Abcam), anti-ROCK1 (1:1,000, #ab134181, Abcam), anti-RhoA (1:500, #ab187027, Abcam), anti-RhoA (phospho S188) (1:1,000, #ab41435, Abcam), and anti-GAPDH (1:1,000, #AB-P-R001, Goodhere Biotech, China). Following primary antibody incubation, membranes were washed with TBST and incubated with secondary antibodies at room temperature for 1 h. Detection of protein bands was performed using enhanced chemiluminescence (Applygen, China), and densitometric analysis of the proteins was carried out using ImageJ software.

### ROS measurement

ROS intracellular concentrations were quantified utilizing the fluorescent probe DCFH-DA (#HY-D0940, Merck Millipore, USA). Cells were treated with a 5 μM solution of DCFH-DA and incubated in darkness at 37 °C for 30 min. Following incubation, cells were rinsed twice with preheated PBS to remove excess probe. Images were rapidly acquired with an Andor Dragonfly 500 confocal microscope (Leica, England), and the fluorescence intensity was quantified by ImageJ software.

### Mitochondrial membrane potential detection

Mitochondrial membrane potential (ΔΨm) was evaluated using the potentiometric dye TMRM (#T668, Invitrogen, USA). Osteoblasts were incubated with a 20 nM solution of TMRM for 30 min at 37 °C, ensuring minimal exposure to light to prevent photobleaching. Following the staining procedure, cells were washed to remove any unbound dye. Fluorescence imaging was conducted expeditiously using an Andor Dragonfly 500 confocal microscope (Leica, England) to capture the mitochondrial accumulation of TMRM. Quantitative analysis of the fluorescence intensity was performed using ImageJ software.

### Seahorse OCR and ECAR measurements

OCR and ECAR measurements were conducted using the XF96 Extracellular Flux Analyzer from Seahorse Bioscience. Cells were plated at a density of 1.0 × 10^4^ cells per well in an XF96 microplate and allowed to adhere overnight. The culture media were replaced with XF media 1 h prior to the assay to standardize conditions. The XF Cell Mito Stress Test Kit was employed to evaluate mitochondrial function in cells, utilizing compounds such as oligomycin, FCCP, antimycin, and rotenone, which were prepared in XF media to final concentrations of 1, 0.5, and 1 μM, respectively. OCR was measured following the manufacturer’s protocol. Additionally, the XF Glycolysis Stress Test Kit was used to assess glycolytic capacity, with glucose, oligomycin, and 2-deoxy glucose (2-DG) being prepared in XF media to final concentrations of 10 mM, 1 μM, and 100 mM, respectively. ECAR was quantified as per the manufacturer’s guidelines.

### Lactate assay

The culture medium was harvested to assess lactate secretion from the cells. Lactate concentrations were quantified utilizing a lactate colorimetric assay kit (#D799851-0050, Sangon Biotech, China), following the manufacturer’s instructions. Absorbance was measured at 450 nm using a BioTek microplate reader and the values were normalized against protein concentration.

### ATP production assay

ATP production in osteoblasts was quantified employing a commercial ATP assay kit (#S0026, Beyotime Biotechnology, China). In strict accordance with the manufacturer’s protocol, osteoblasts were lysed using the kit-provided lysate buffer. The cell lysates from various groups were then subjected to centrifugation at 12,000 *g* for 5 min at 4 °C to procure the supernatants. ATP levels in the supernatants were subsequently measured using a Spark luminometer (Tecan, Switzerland). To account for variations in cell number and size, ATP concentrations were normalized against the total protein content determined for each well.

### siRNA transfection and drug treatment

Gene silencing in osteoblasts was achieved by transfecting cells with small interfering RNAs (siRNAs) targeting Drp1 and YAP, which were custom-designed and synthesized by GenePharma Corporation (Shanghai, China). Transfection was performed using the Lipo3000 reagent (#L3000008, Thermo Fisher Scientific), following the manufacturer’s instructions. Control transfections employed a scrambled, nontargeting siRNA sequence (siCon). The specific siRNA sequences used were as follow: siYAP, 5′-GACATCTTCTGGTCAAAGA-3′; siDrp1, 5′-GCAGAACUCUAGCUGUAAUTT-3′; and siCon, 5′-GACATCTTCTGGTCAAAGA-3′. The effectiveness of the silencing was confirmed through Western blot analysis.

To elucidate the contributions of mitochondrial fission, mechanotransduction, and epigenetic modification to osteoblast function under SMG, cells underwent treatment with targeted pharmacological modulators concurrent with SMG exposure. Mitochondrial fission was inhibited using mdivi-1 (#M0199, Sigma-Aldrich). Substrate adhesion disruption was achieved with 1.25 mM EDTA (#E9884, Sigma-Aldrich), and Rho kinase (ROCK) activity was inhibited using 10 μM Y27632 (#Y0503, Sigma-Aldrich). Furthermore, to target epigenetic modifications, H3K9me3 and H3K27me3 were inhibited using 5 μM chaetocin (#S8068, Selleckchem), a specific inhibitor of the H3K9 methyltransferase Su(var)3–9, and 5 μM GSK343 (#S7164, Selleckchem), a specific inhibitor of the H3K27 methyltransferase EZH2, respectively.

### Immunofluorescence staining

Osteoblasts were fixed in 4% PFA for 20 min at room temperature and washed thrice with PBS. For permeabilization, cells were treated with 0.2% Triton X-100 for 5 min. After additional PBS washes, nonspecific sites were blocked with 5% BSA for 2 h at room temperature. The cells were subsequently incubated overnight at 4 °C with primary antibodies against paxillin (1:200, Abcam #ab32084), YAP (1:200, Cell Signaling Technology #14074), Myosin II (1:200, Sigma-Aldrich #M8064), H3K9me3 (1:500, Cell Signaling Technology #13969), and H3K27me3 (1:800, Cell Signaling Technology #9733). Secondary detection involved Alexa Fluor 488-labeled antibodies (1:200, Abcam) incubated at 37 °C for 1 h. F-actin, G-actin, and nuclei were visualized using Texas red isothiocyanate-conjugated phalloidin (1:800, Thermo Fisher Scientific #A34055), DNase I 488 (1:500, Thermo Fisher Scientific #D12371), and DAPI (1:2,000, Sigma-Aldrich #D9542), respectively. Cells were washed thrice gently using PBS and fixed with 4% PFA at room temperature for 20 min. Fluorescent images were acquired using a TCS-SP8 confocal microscope or an Andor Dragonfly 500 confocal laser-scanning microscope. Z-stack confocal 3D images were captured at 0.15-μm intervals using the confocal laser-scanning microscope and analyzed with IMARIS software for detailed 3D structural examination.

For super-resolution imaging, cells were incubated overnight at 4 °C with a primary antibody against TOM20 (1:200, Santa Cruz Biotechnology #sc-17764). The secondary antibody was Abberior STAR RED (1:150, Abberior). Super-resolution images were obtained using a STED super-resolution microscope (Abberior Instruments, Germany) equipped with an Olympus 100×, numerical aperture 1.45 objective lens. Mitochondrial morphology was quantified using Fiji software (MiNA and Fiji plugin, http://fiji.sc/Fiji). ImageJ software was employed for additional image analyses.

### Calculation of the CCP

Chromatin condensation was assessed by first fixing the cells in 4% PFA for 20 min at room temperature. The cells were then washed and permeabilized with a 0.2% Triton X-100 solution in PBS for 5 min. For nuclear visualization, DAPI staining was conducted, and the mid-section of the nuclei was imaged using a TCS-SP8 confocal microscope (Leica Microsystems, Austria). The CCP was determined by applying a gradient-based Sobel edge detection algorithm within MATLAB to quantify edge density in individual nuclei [59]. This computational analysis enables the measurement of chromatin compaction by evaluating the intensity and distribution of edges in the nuclear images.

### Statistical analysis

Statistical representations in all figures are expressed as mean ± SD, derived from no fewer than 3 independent biological replicates. Statistical evaluations were conducted utilizing SPSS software (version 13.0; SPSS Inc., USA). The distribution of the data was assessed for normality using the Shapiro–Wilk test. Determination of statistical significance was based on the appropriate test contingent on data normality. For normally distributed data, comparisons between 2 groups were made using an unpaired 2-tailed Student’s *t* test, whereas for multiple group comparisons, one-way ANOVA was employed, followed by Tukey’s post-hoc multiple comparison test. In cases where the normality criterion was not met, equivalent nonparametric tests were applied. A threshold of *P* < 0.05 was set for establishing statistically significant differences. Explicit details regarding sample sizes and specific levels of significance are elucidated within the figure legends.

## Data Availability

All data needed to evaluate the conclusions in the paper are present in the paper and/or the Supplementary Materials.
