# Supplementary material for: Inhibition of Mitochondrial Fission Reverses Simulated Microgravity-Induced Osteoblast Dysfunction by Enhancing Mechanotransduction and Epigenetic Modification
Source: Research (Wash D C). 2025 Feb 4;8:0602. doi: 10.34133/research.0602 (PMC11791006; doi:10.34133/research.0602)
Supplement: Supplementary 1 — Figs. S1 to S15 Table S1 [file research.0602.f1.docx]

**Supplementary materials**

**Inhibition of Mitochondrial Fission Reverses Simulated Microgravity-induced Osteoblast Dysfunction by Enhancing Mechanotransduction and Epigenetic Modification**

Qiusheng Shi^1†^, Yaxin Song^1†^, Jingqi Cao^1^, Jing Na^1^, Zhijie Yang^1^, Xinyuan Chen^1^, Ziyi Wang^1^, Yubo Fan^1*^, Lisha Zheng^1*^

^1^Key Laboratory of Biomechanics and Mechanobiology (Beihang University), Ministry of Education, Beijing Advanced Innovation Center for Biomedical Engineering, School of Biological Science and Medical Engineering, Beihang University, Beijing, 100083, China.

***Corresponding author.** email: [yubofan@buaa.edu.cn](mailto:yubofan@buaa.edu.cn), [lishazheng@buaa.edu.cn](mailto:lishazheng@buaa.edu.cn)

**This Word file includes:**

Supplementary Text

Fig. S1 to S15

Table. S1

**
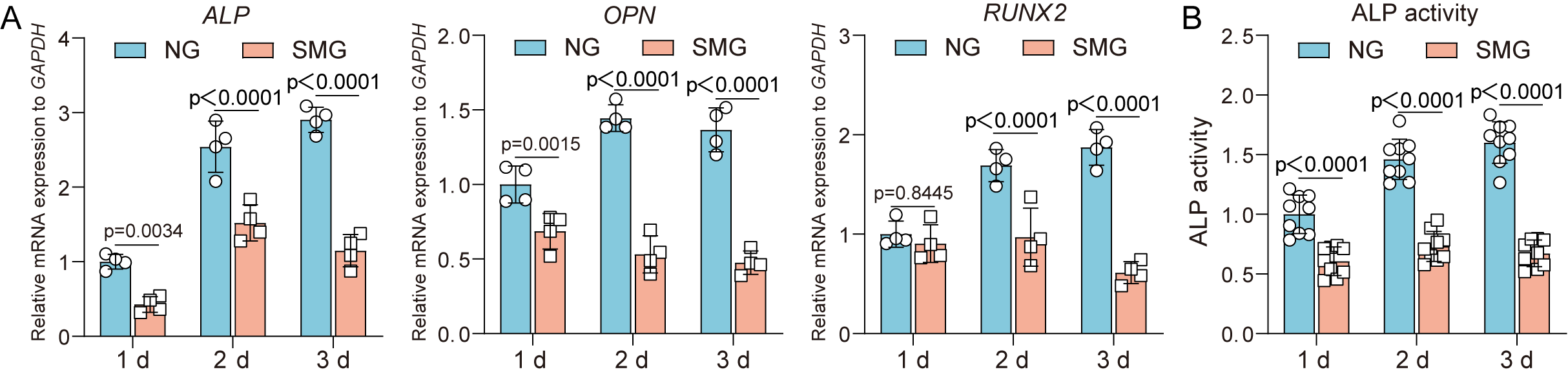
**

**Fig. S1.** (A) Altered gene expression of osteoblast markers *ALP*, *OPN*, and *RUNX2* after 1–3 days under NG or SMG conditions (n ≥ 3). (B) Suppressed ALP enzyme activity in osteoblasts after 1–3 days under NG or SMG conditions (n = 9 for each group). Results reflect ≥ 3 biological repeats, mean ± SD, *p*-values via ANOVA or t-test.

**
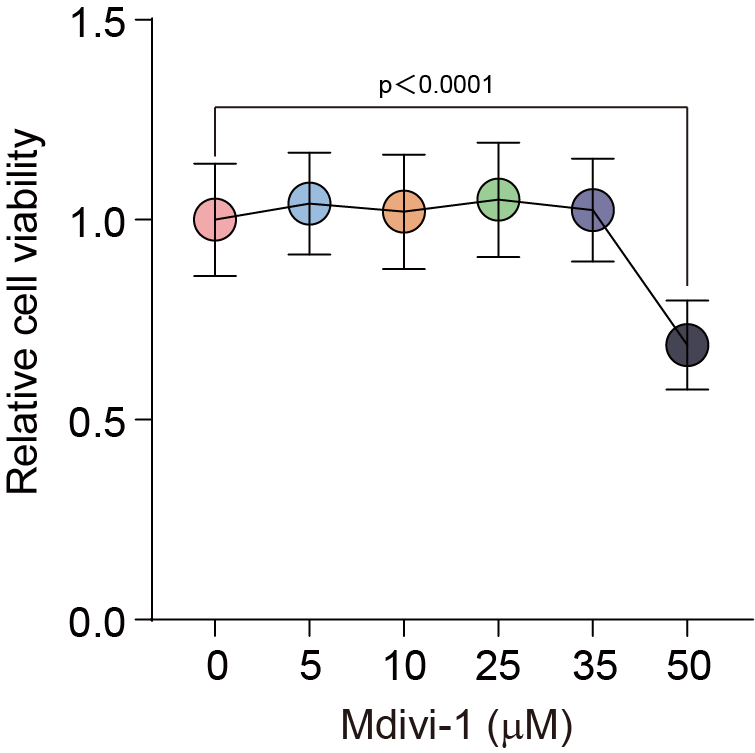
**

**Fig. S2.** Effect of mdivi-1 concentration on osteoblast viability. Osteoblasts were subjected to increasing concentrations of Mdivi-1 (0, 5, 10, 25, 35, and 50 µM). Cellular viability was evaluated 72 hours after exposure using the CCK-8 assay (n = 11 for each group). Results reflect ≥ 3 biological repeats, mean ± SD, *p*-values via ANOVA or t-test.

**
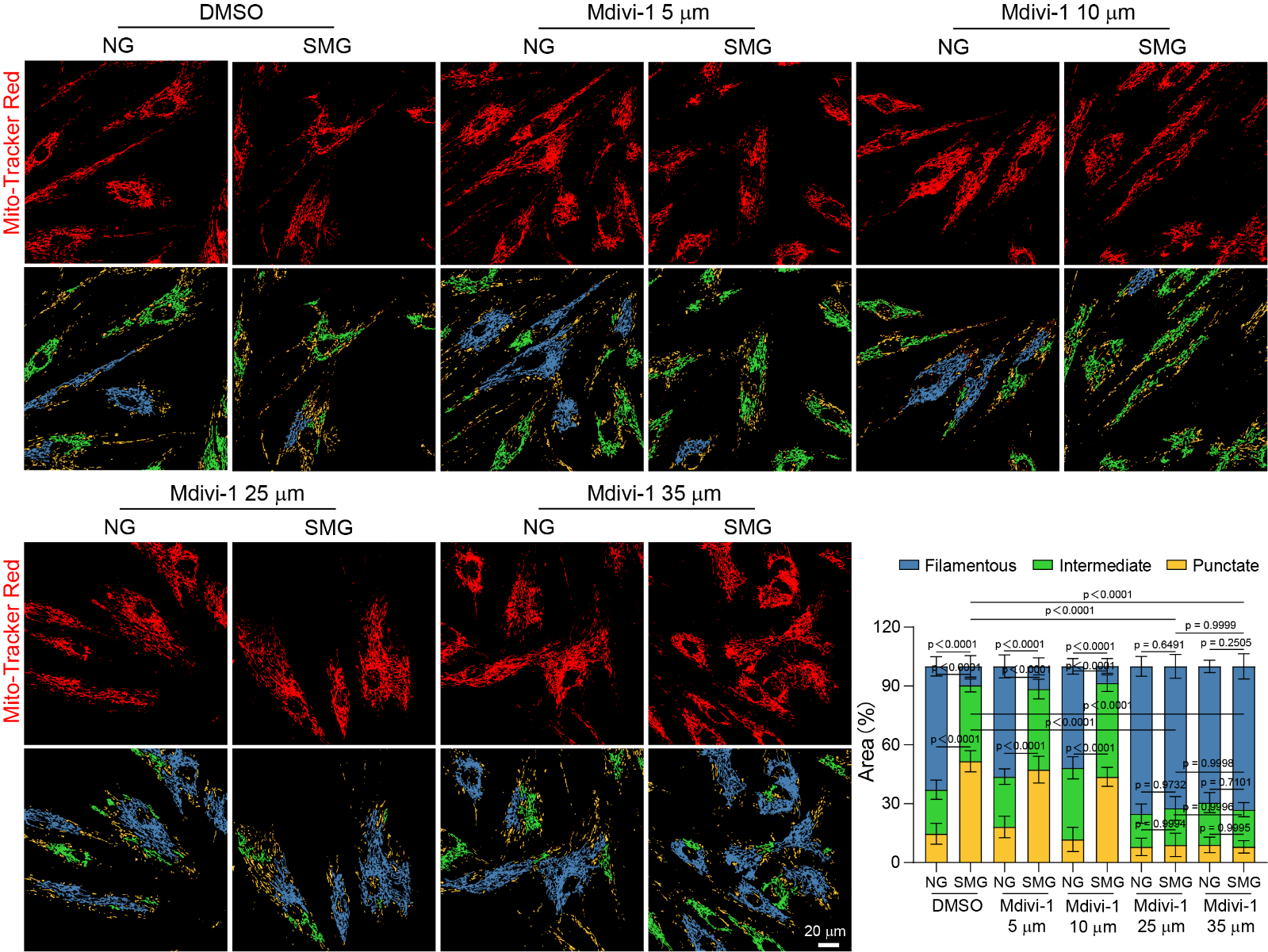
**

**Fig. S3.** Effect of mdivi-1 concentration on mitochondrial morphology in osteoblasts under NG and SMG conditions. Osteoblasts were treated with DMSO or mdivi-1 at concentrations of 5, 10, 25, and 35 µM, following 3 days of exposure to NG or SMG. Representative immunofluorescent images depicting osteoblast mitochondria labeled with MitoTracker Red, categorized by mitochondrial morphology and displayed with various colors under NG or SMG conditions. Scale bars: 20 μm. Classification of mitochondria based on the area percentages of punctate, intermediate, and filamentous structures (n = 25 for each group). Results reflect ≥ 3 biological repeats, mean ± SD, *p*-values via ANOVA or t-test.


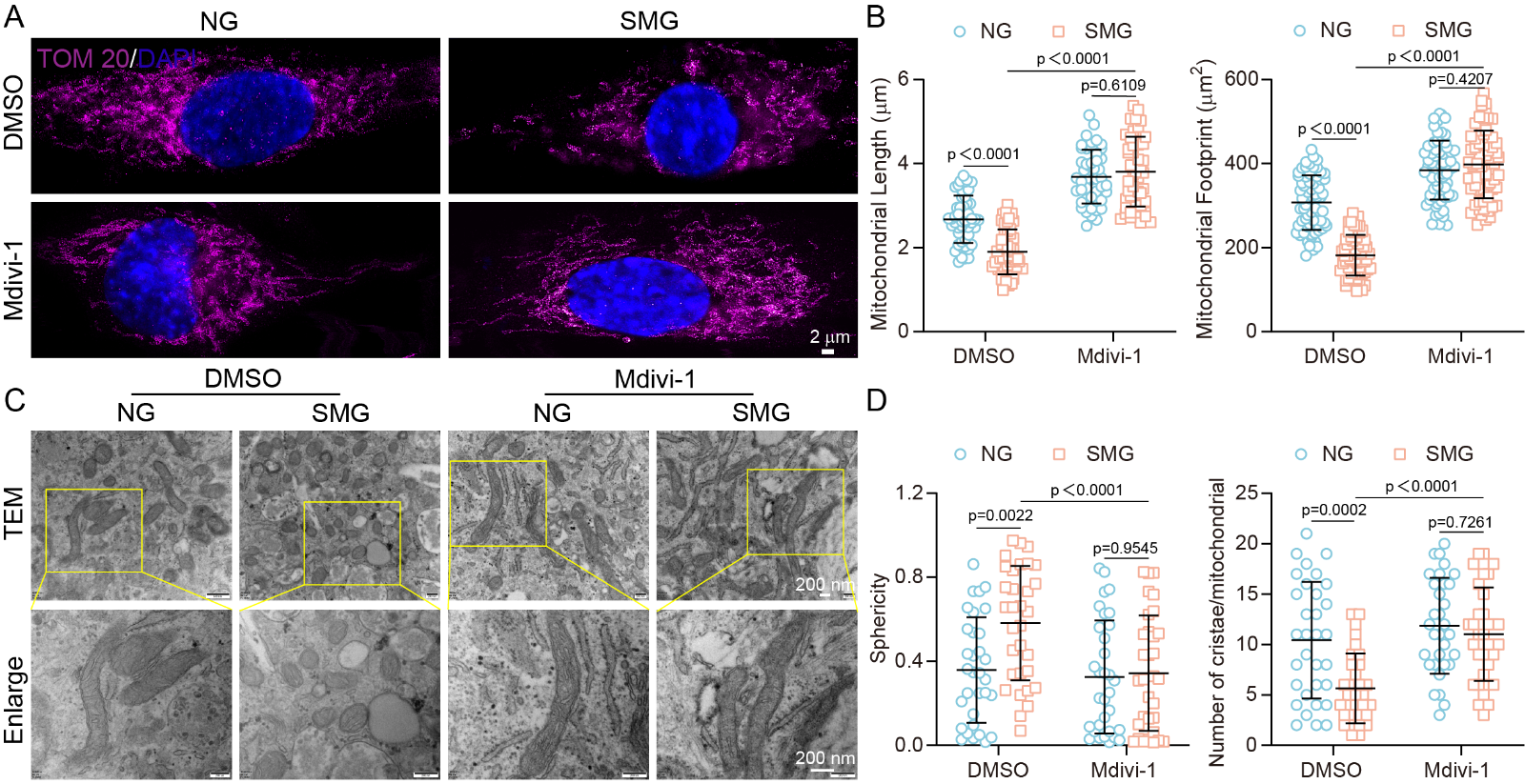


**Fig. S4.** Mdivi-1 protection against SMG-induced mitochondrial fission in osteoblasts. Osteoblasts subjected to treatment with mdivi-1 (25 μM) after 3 days of NG or SMG exposure. (A) Super-resolution STED imaging was used to visualize mitochondria labeled with TOM20, scale bars: 2 μm. (B) Metrics of mitochondrial length (n = 48 for each group) and footprint (n = 51 for each group). (C) TEM analysis of mitochondrial ultrastructure. Yellow boxes indicate the regions that were shown as a magnified view, scale bars: 200 nm. (D) Quantitative assessment of mitochondrial sphericity and cristae number (n = 32 for each group). Results reflect ≥ 3 biological repeats, mean ± SD, *p*-values via ANOVA or t-test.


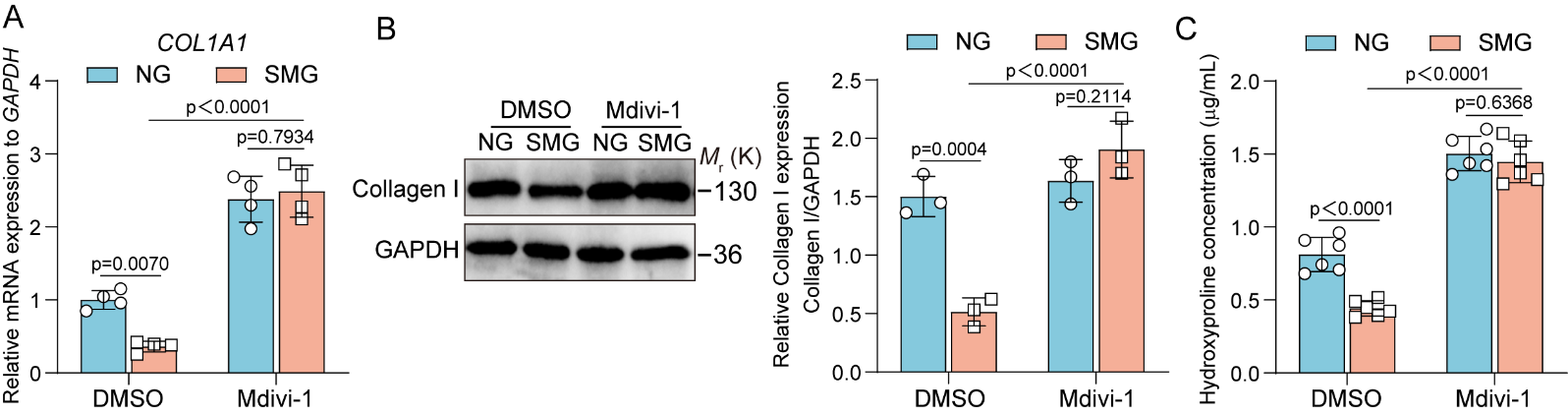


**Fig. S5.** Inhibition of mitochondrial fission reverses SMG-induced reduction in collagen levels. Osteoblasts subjected to treatment with mdivi-1 (25 μM) after 3 days of NG or SMG exposure. (A) Gene expression of *COL1A1* (n ≥ 3). (B) The representative images and quantitative analysis of the collagen Ⅰ protein level expression (n = 3). (C) Hydroxyproline assay (n = 6 for each group). Results reflect ≥ 3 biological repeats, mean ± SD, *p*-values via ANOVA or t-test.


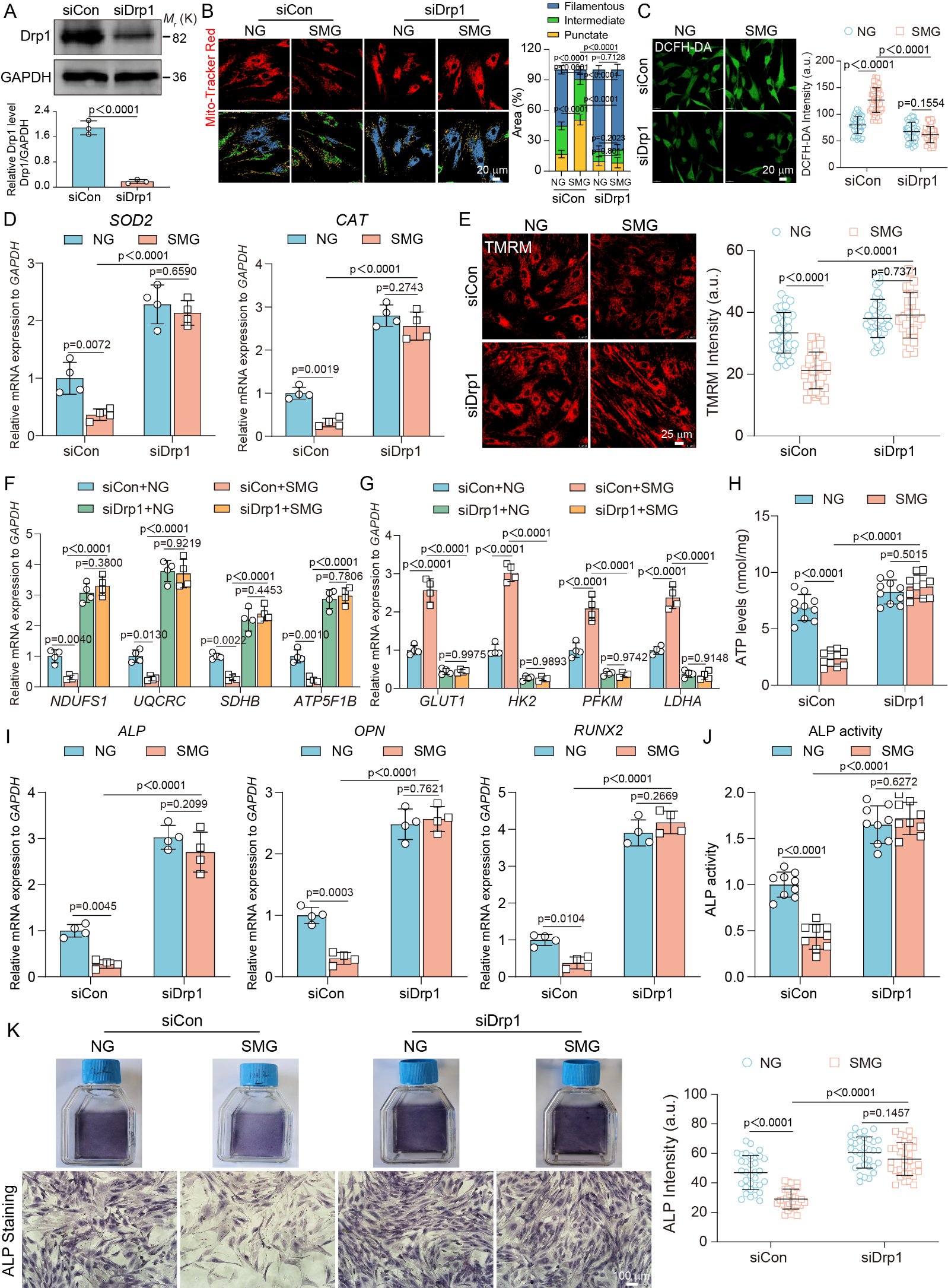


**Fig. S6.** Knockdown of Drp1 rescues SMG-induced osteoblasts dysfunction. (A) Lysates of osteoblasts transfected with control siRNA (siCon) or Drp1 siRNA (siDrp1) were subjected to western blot analyses with corresponding antibodies. Representative images and quantitative analysis of Drp1 protein level expression (n = 3). Osteoblasts transfected with siCon or siDrp1 after 3 days of NG or SMG exposure. (B) Representative immunofluorescent images depicting osteoblast mitochondria labeled with Mito-Tracker Red, categorized by mitochondrial morphology and displayed with various colors under NG or SMG conditions, Scale bars: 20 μm. Classification of mitochondria based on the area percentages of punctate, intermediate, and filamentous structures (n = 23 for each group). (C) DCFH-DA staining for ROS, scale bars: 20 μm. Quantification of ROS intensity (n = 68 for each group). (D) Gene expression of *SOD2* and *CAT* (n ≥ 3). (E) TMRM staining for ΔΨm, scale bars: 25 μm. Quantification of ΔΨm intensity (n = 38 for each group). (F, G) Gene expression of oxidative phosphorylation family genes (*NDUFB8*, *UQCRC2*, *SDHB*, and *ATP5F1B*) and glycolysis family genes (*GLUT1*, *HK2*, *PFKM*, and *LDHA*) in osteoblasts (n ≥ 3). (H) ATP levels (n = 10 for each group). (I) Gene expression of *ALP*, *OPN*, and *RUNX2* (n ≥ 3). (J, K) ALP activity (n = 9 for each group) and ALP staining and intensity (n = 34 for each group), scale bars: 100 μm. Results reflect ≥ 3 biological repeats, mean ± SD, *p*-values via ANOVA or t-test.

**
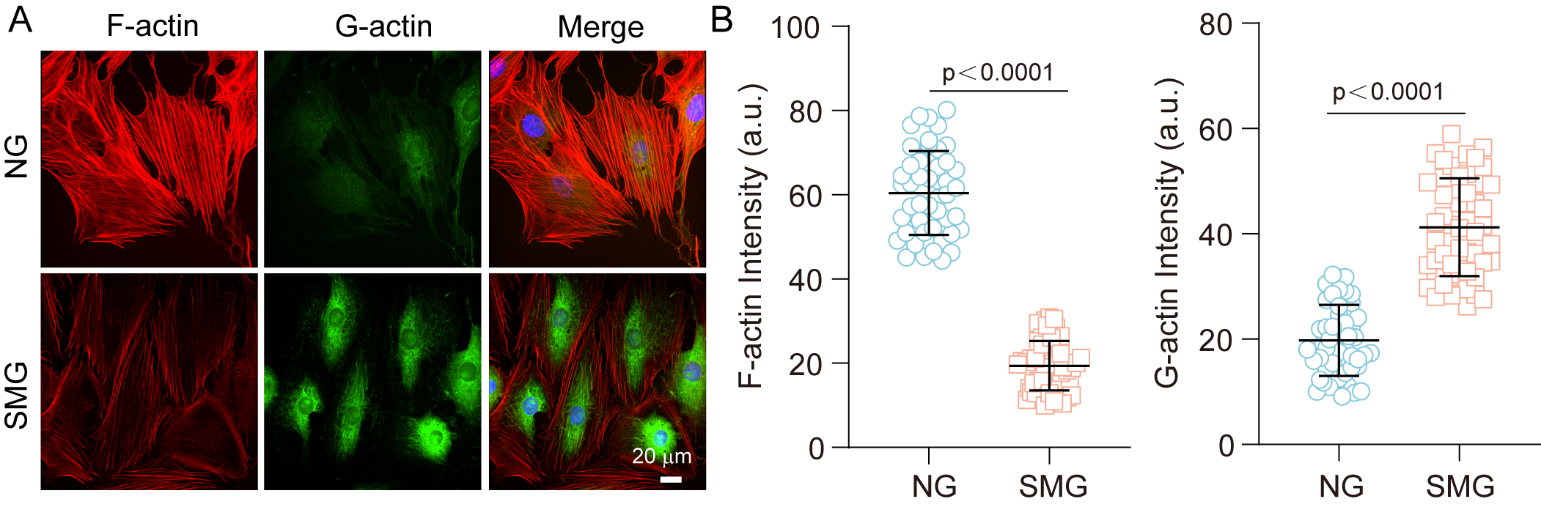
**

**Fig. S7.** (A) Osteoblasts were fixed and immunostained using rhodamine phalloidin for F-actin (red), DNase I 488 for G-actin (green), and DAPI for nuclei (blue) after 3 days of NG or SMG exposure. Scale bars: 20 μm. (B) F-actin and G-actin intensity quantifications (n = 51 for each group). Results reflect ≥ 3 biological repeats, mean ± SD, *p*-values via ANOVA or t-test.

**
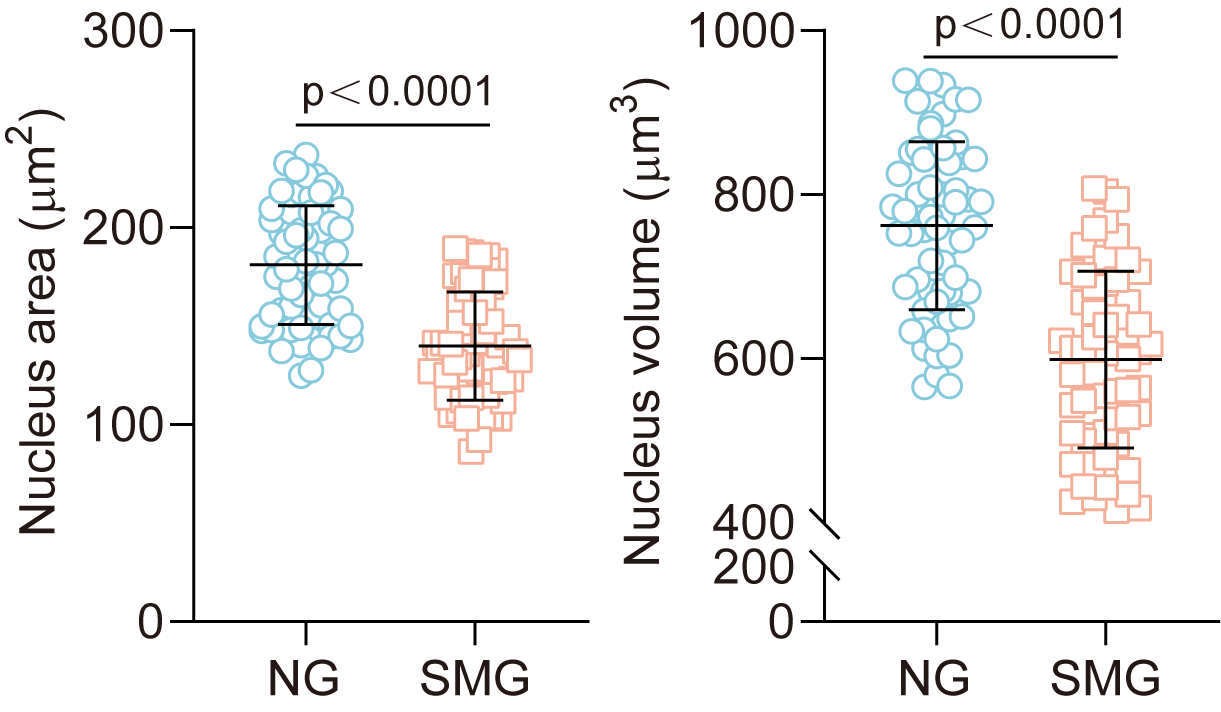
**

**Fig. S8.** The quantitative analysis examined the nuclear area projected onto the XY plane and the volume of nuclei reconstructed in 3D following 3 days of exposure to NG or SMG (n = 62 for each group). Results reflect ≥ 3 biological repeats, mean ± SD, *p*-values via ANOVA or t-test.


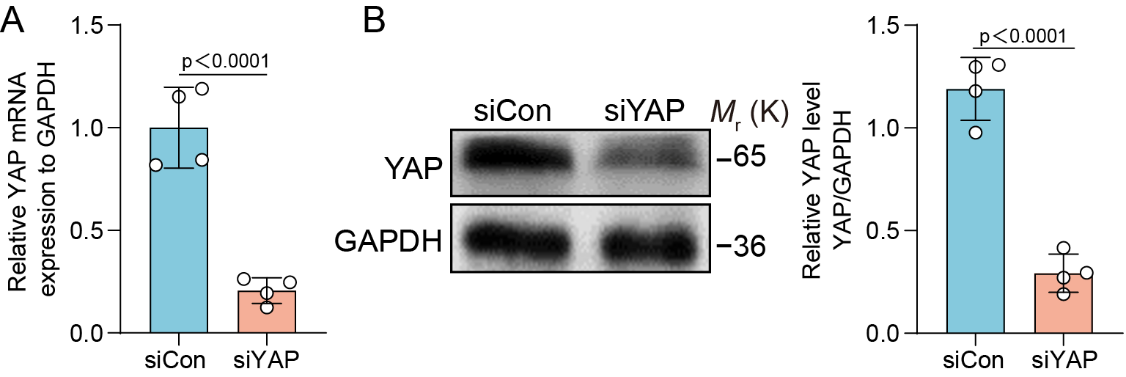


**Fig. S9.** Assessment of YAP knockdown efficiency in osteoblasts. (A) Relative gene expression of YAP analyzed by qPCR in osteoblasts transfected with control siRNA (siCon) or YAP siRNA (siYAP) (n ≥ 3). (B) Lysates of osteoblasts transfected with siCon or siYAP were subjected to western blot analyses with corresponding antibodies. The panels display the relative protein expression in osteoblasts (n ≥ 3). Results reflect ≥ 3 biological repeats, mean ± SD, p-values via ANOVA or t-test.


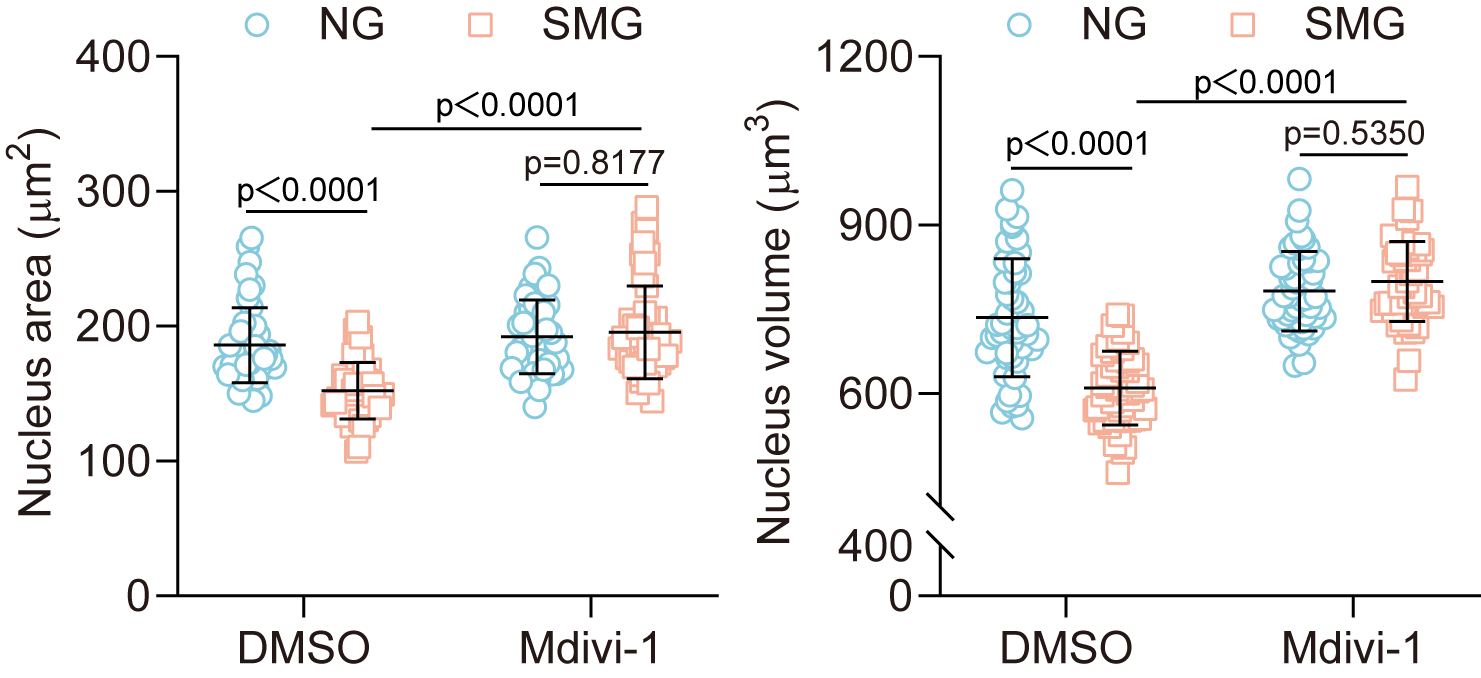


**Fig. S10.** Osteoblasts treated with the DMSO or mitochondrial fission inhibitor mdivi-1 (25 μM) after 3 days of NG or SMG exposure. The quantitative analysis examined the nuclear area projected onto the XY plane and the volume of nuclei reconstructed in 3D (n = 48 for each group). Results reflect ≥ 3 biological repeats, mean ± SD, *p*-values via ANOVA or t-test.


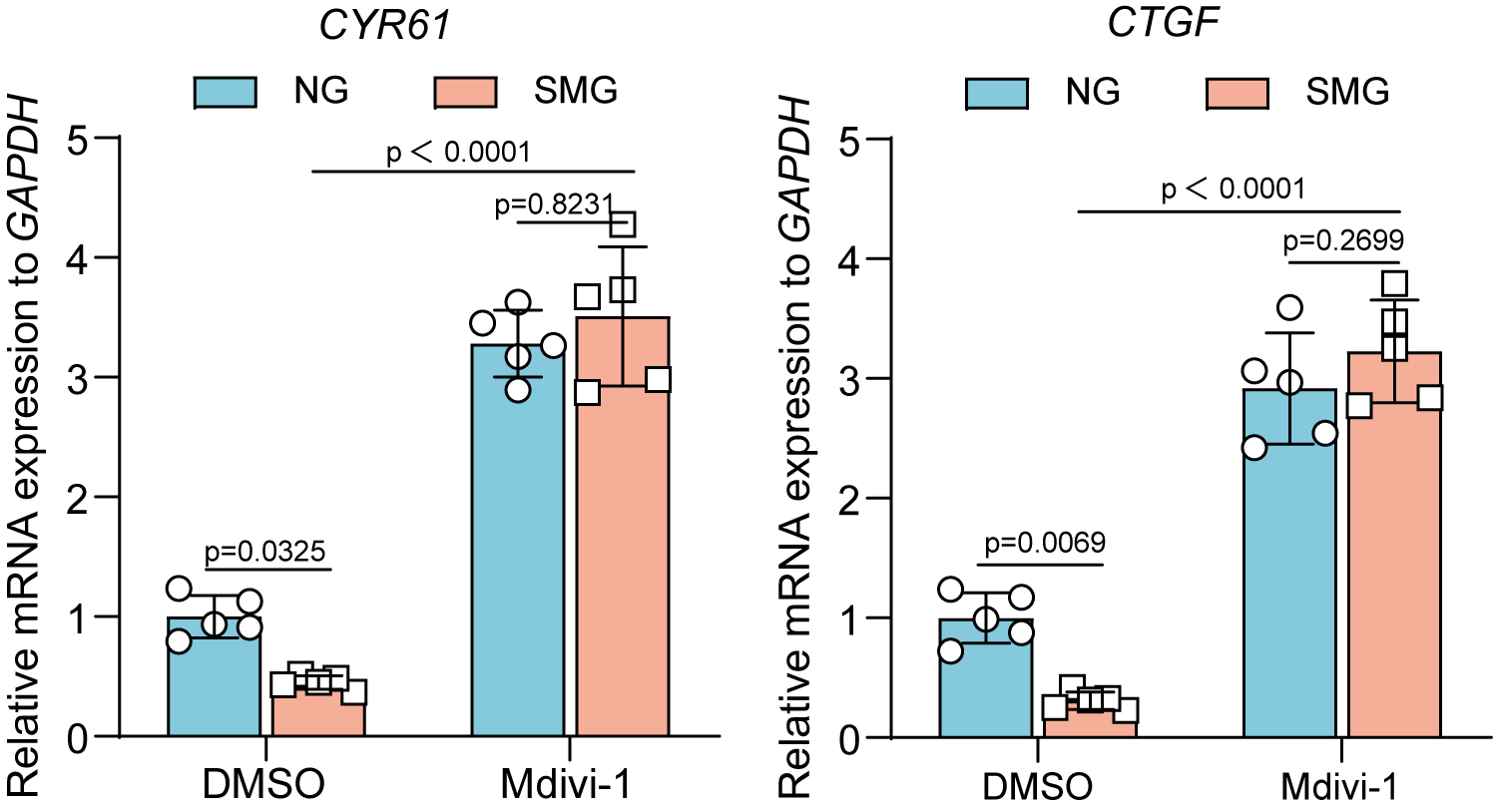


**Fig. S11.** Osteoblasts treated with the DMSO or mitochondrial fission inhibitor mdivi-1 (25 μM) after 3 days of NG or SMG exposure. The gene expression of *CYR61* and *CTGF* (n ≥ 3). Results reflect ≥ 3 biological repeats, mean ± SD, *p*-values via ANOVA or t-test.


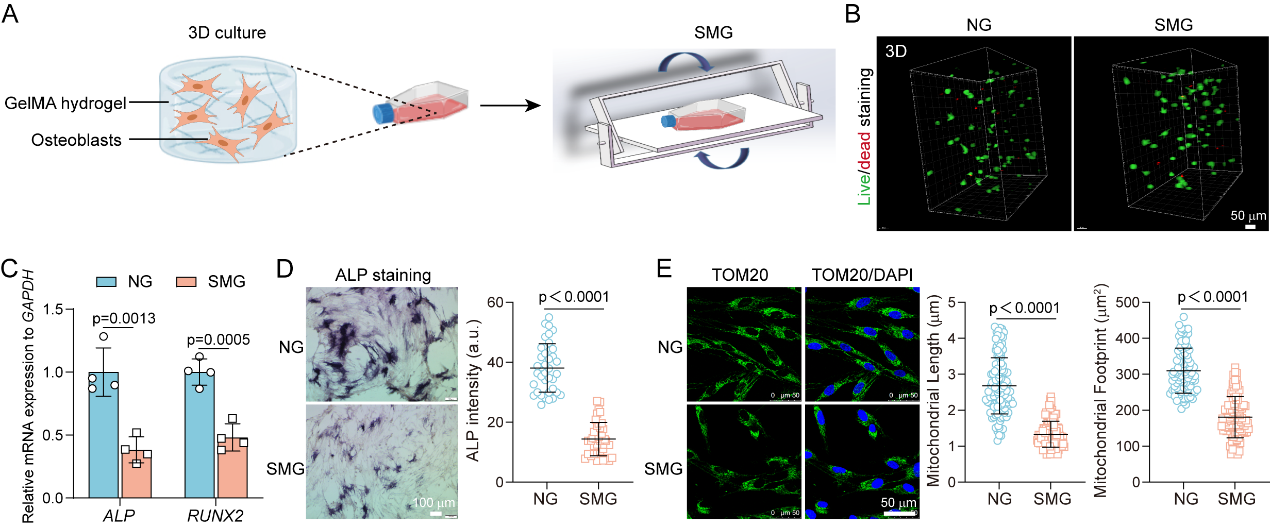


**Fig. S12.** SMG induces osteoblast dysfunction and mitochondrial fission in 3D culture conditions. (A) Schematic of the preparation process for osteoblast-laden GelMA hydrogels used in 3D cultures for SMG investigations. (B) Viability assay showing live/dead staining of osteoblasts after 3 days under NG and SMG conditions. scale bars: 50 μm. (C) Changes in the gene expression of osteoblast markers *ALP* and *RUNX2* after 3 days under NG and SMG conditions (n ≥ 3). (D) ALP staining and quantification of staining intensity after 3 days under NG or SMG conditions (n ≥ 38), scale bar: 100 μm. (E) Immunofluorescent images of osteoblast mitochondria labeled with TOM20 after 3 days under NG or SMG conditions, scale bars: 50 μm. Metrics of mitochondrial length (n ≥ 114) and footprint (n ≥ 88). Results reflect ≥ 3 biological repeats, mean ± SD, *p*-values via t-test.


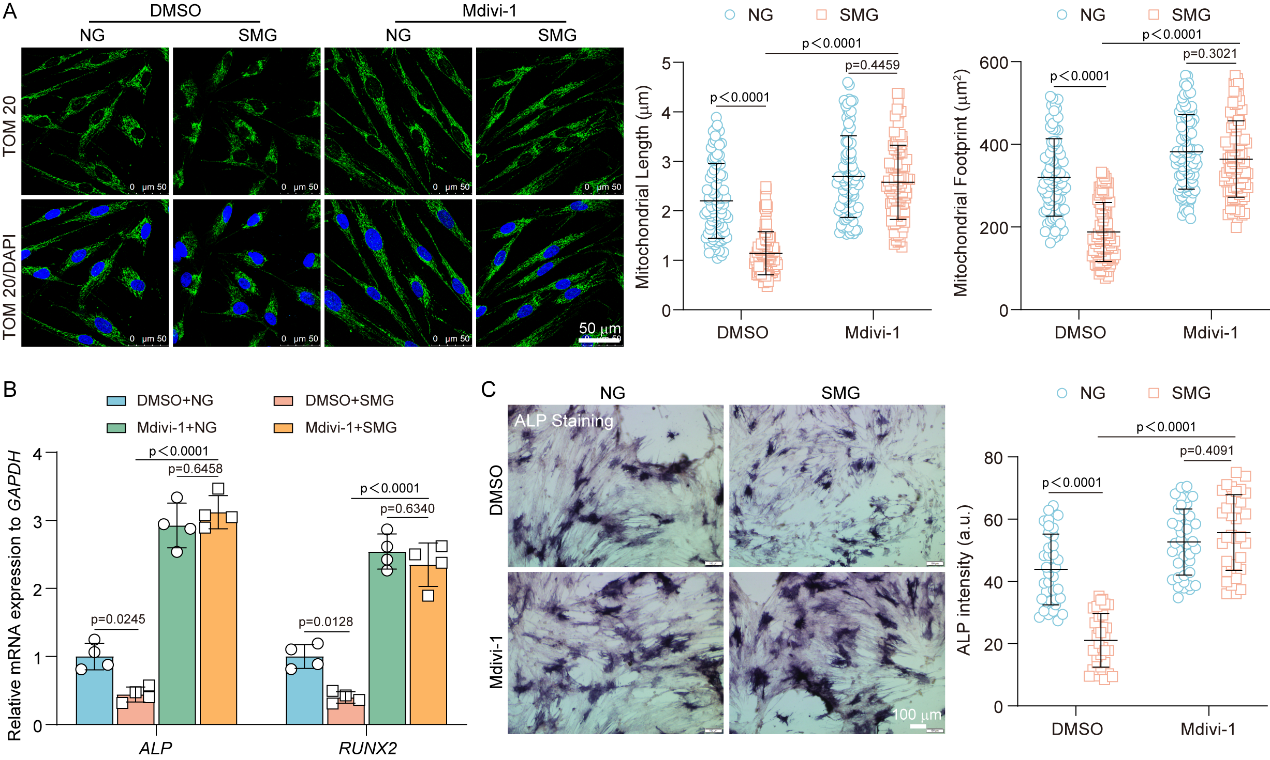


**Fig. S13.** Inhibition of mitochondrial fission restores osteoblast function under SMG in 3D Culture. (A) Osteoblasts treated with 25 µM mdivi-1 after 3 days of exposure to NG or SMG in 3D culture. Immunofluorescent staining was used to visualize mitochondria labeled with TOM20, scale bars: 50 µm. Metrics of mitochondrial length (n ≥ 94) and footprint (n ≥ 86). (B) Gene expression of *ALP* and *RUNX2* (n ≥ 3). (C) ALP staining and intensity (n ≥ 33), scale bars: 100 μm. Results reflect ≥ 3 biological repeats, mean ± SD, p-values via ANOVA or t-test.

**
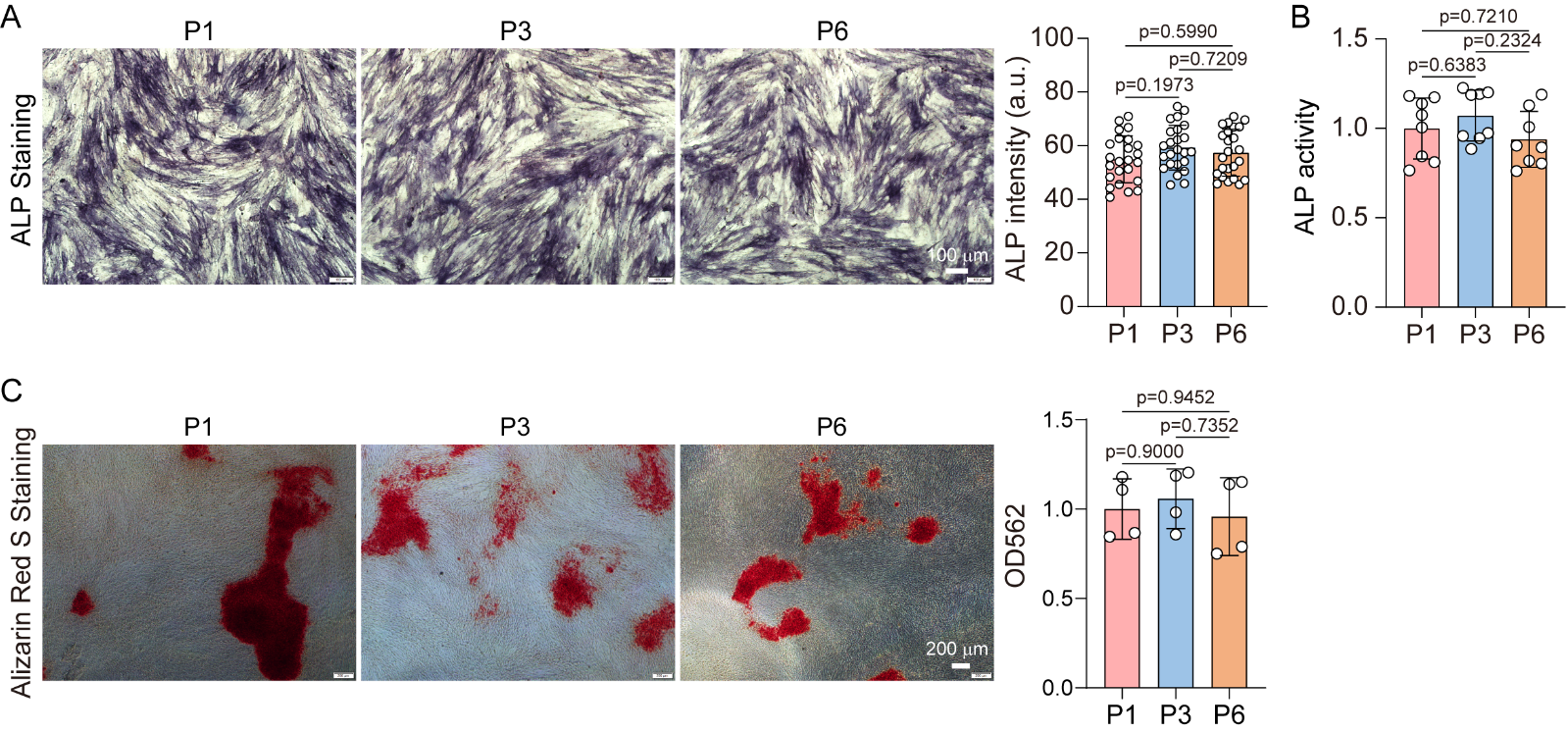
**

**Fig. S14.** Assessment of osteoblastic phenotype stability across different passages (P1, P3, P6). (A) ALP staining of osteoblasts at passages P1, P3, and P6. Scale bar: 100 µm. Quantitative analysis of ALP intensity shows no significant differences among the passages (n = 24 for each group). (B) ALP activity assay of osteoblasts at passages P1, P3, and P6, indicating no significant differences in enzymatic activity (n = 8 for each group). (C) Osteoblasts from passages P1, P3, and P6 were cultured for 14 days and stained with Alizarin S Red. Scale bar: 200 µm. Quantification of Alizarin Red S staining demonstrates no significant differences in mineral deposition across the passages (n = 4 for each group). Results reflect ≥ 3 biological repeats, mean ± SD, *p*-values via ANOVA or t-test.

**
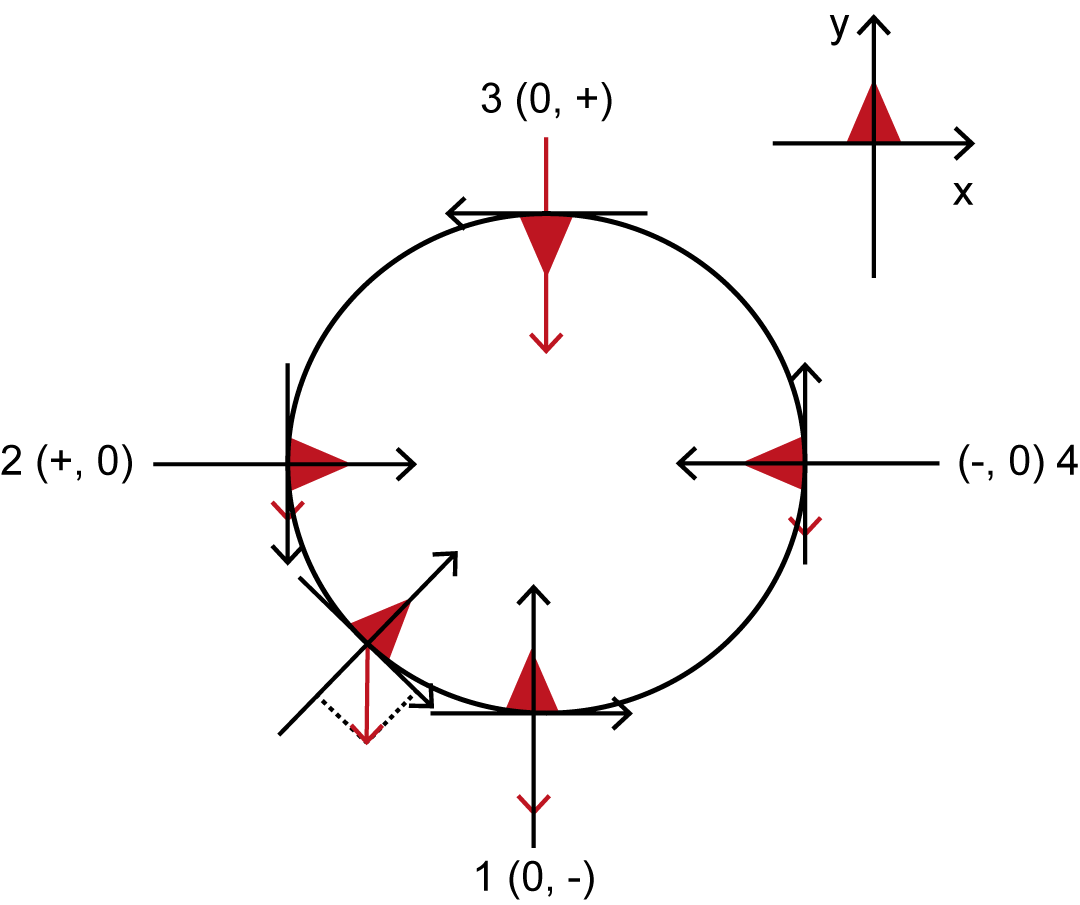
**

**Fig. S15.** Principles of SMG with a two-dimensional clinostat. The clinostat's principle for modeling SMG is clarified as follows: Cells are attached to a slide; when the clinostat rotates the sample by 90° (from position 1 to position 2), the direction of the gravitational force shifts from (0, -) to (0, +). Through a full 360° rotation, the average gravitational force experienced by the cells effectively becomes approximately 0 g. This renders the cells unable to perceive gravity, effectively bypassing their gravitational detection mechanisms.

**Table. S1.** Primers used for RT-qPCR.

| Gene symbol | Protein | GenBank accession no. | Sequence (5’ - 3’) |
| --- | --- | --- | --- |
| *ALP* | Alkaline phosphatase | NM_013059.2 | F: GTGTTGTACGTCTTGGAGAGA |
|  |  |  | R: CATGTTCCTGGGAGATGGTA |
| *OPN* | Osteopontin | NM_012881.2 | F: GACGGCCGAGGTGATAGCTT |
|  |  |  | R: CATGGCTGGTCTTCCCGTTGC |
| *RUNX2* | Runt-related transcription factor 2 | NM_001278484.3 | F: GCCGGGAATGATGAGAACTA |
|  |  |  | R: GGACCGTCCACTGTCACTTT |
| *COL1A1* | Collagen type I alpha 1 | NM_053304.1 | F: GAGCGGAGAGTACTGGATCGA |
|  |  |  | R: CTGACCTGTCTCCATGTTGCA |
| *SOD2* | Superoxide dismutase 2 | NM_017051.2 | F: TTCAGCCTGCACTGAAG |
|  |  |  | R: GTCACGCTTGATAGCCTC |
| *CAT* | Catalase | NM_012520.2 | F: ATGGCTTTTGACCCAAGCAA |
|  |  |  | R: CGGCCCTGAAGCTTTTTGT |
| *NDUFB8* | NADH: ubiquinone oxidoreductase subunit B8 | NM_001005550.1 | F: TTTCTAGAGGGGAAGCGTGC |
|  |  |  | R: TGCAGCGGGTACACTGTATG |
| *UQCRC2* | Ubiquinol-cytochrome c reductase core protein 2 | NM_001006970.1 | F: CCGGGTCCTTCTCGAGATTTTAT |
|  |  |  | R: AACTCAAGTTCCTGAGGCTGC |
| *SDHB* | Succinate dehydrogenase complex iron sulfur subunit B | NM_001100539.1 | F: TCGCCATTTACCGATGGGAC |
|  |  |  | R: GCACCATCGGTCCACACTTAT |
| *ATP5F1B* | ATP synthase F1 subunit beta | NM_134364.1 | F: GCATTTAGGGGAGAGCACCG |
|  |  |  | R: TTTTGATTGGTGCCCCCGAA |
| *GLUT1* | Glucose transporter type 1 | NM_138827.1 | F: AGCCCCGAGGATCTCTCTGG |
|  |  |  | R: GATCTGAGCAACAGTCTTGC |
| *HK2* | Hexokinase type 2 | NM_012735.2 | F: GCAGCTGTGAAAATGTTGCC |
|  |  |  | R: CCGTCACCCTTACTCGGAGC |
| *PFKM* | Phosphofructokinase type M | NM_031715.2 | F: CCAAGGCAACCGAGTGCTGG |
|  |  |  | R: GGTTCTTCTTGGGTAGAGTCC |
| *LDHA* | Lactate dehydrogenase A | NM_017025.2 | F: TGGCCTGTGCCATCAGTATC |
|  |  |  | R: TTCCAAGCCACGTAGGTCAA |
| *CYR61* | Cysteine-rich 61 | NM_031327.3 | F: AGACCAGGACCGTGAAGATG |
|  |  |  | R: TCCGACCCACACTAGAAACC |
| *CTGF* | Connective tissue growth factor | NM_022266.2 | F: CTGCCATTACAACTGTCCCG |
|  |  |  | R: GTCCCTTACTCCCTGGCTTT |
| *YAP* | Yes-associated protein | NM_001394328.1 | F: CCGGGATGTCTCAGGAATTG |
|  |  |  | R: CTGTAGCTGCTCATGCTTAGTCCA |
| *GAPDH* | Glyceraldehyde-3-phosphate dehydrogenase | NM_017008.4 | F: TGTGTCCGTCGTGGATCTGA |
|  |  |  | R: TTGCTGTTGAAGTCGCAGGAG |
